# *KCTD*: A new gene family involved in neurodevelopmental and neuropsychiatric disorders

**DOI:** 10.1111/cns.13156

**Published:** 2019-06-14

**Authors:** Xinchen Teng, Abdel Aouacheria, Loïc Lionnard, Kyle A. Metz, Lucian Soane, Atsushi Kamiya, J. Marie Hardwick

**Affiliations:** ^1^ Jiangsu Key Laboratory of Neuropsychiatric Diseases and College of Pharmaceutical Sciences Soochow University Suzhou China; ^2^ W. Harry Feinstone Department of Molecular Microbiology and Immunology Johns Hopkins University Bloomberg School of Public Health Baltimore Maryland; ^3^ ISEM, Institut des Sciences de l'Evolution de Montpellier, CNRS, EPHE, IRD Université de Montpellier Montpellier France; ^4^ Department of Psychiatry and Behavioral Sciences Johns Hopkins School of Medicine Baltimore Maryland; ^5^ Present address: Feinberg School of Medicine Northwestern University Chicago USA

**Keywords:** KCTD11, KCTD13, KCTD7, Neurodegeneration, Neurodevelopmental disorders

## Abstract

The underlying molecular basis for neurodevelopmental or neuropsychiatric disorders is not known. In contrast, mechanistic understanding of other brain disorders including neurodegeneration has advanced considerably. Yet, these do not approach the knowledge accrued for many cancers with precision therapeutics acting on well‐characterized targets. Although the identification of genes responsible for neurodevelopmental and neuropsychiatric disorders remains a major obstacle, the few causally associated genes are ripe for discovery by focusing efforts to dissect their mechanisms. Here, we make a case for delving into mechanisms of the poorly characterized human *KCTD* gene family. Varying levels of evidence support their roles in neurocognitive disorders (*KCTD3*), neurodevelopmental disease (*KCTD7*), bipolar disorder (*KCTD12*), autism and schizophrenia (*KCTD13*), movement disorders (*KCTD17*), cancer (*KCTD11*), and obesity (*KCTD15*). Collective knowledge about these genes adds enhanced value, and critical insights into potential disease mechanisms have come from unexpected sources. Translation of basic research on the KCTD‐related yeast protein Whi2 has revealed roles in nutrient signaling to mTORC1 (KCTD11) and an autophagy‐lysosome pathway affecting mitochondria (KCTD7). Recent biochemical and structure‐based studies (KCTD12, KCTD13, KCTD16) reveal mechanisms of regulating membrane channel activities through modulation of distinct GTPases. We explore how these seemingly varied functions may be disease related.

## 1 INTRODUCTION

Understanding the molecular basis of neurodevelopmental and neuropsychiatric disorders has many obstacles inherent to disease complexities and the lack of tractable model systems analogous to cancer biology. However, remarkable advancements in genomics have identified many potential candidate genes, some with additional compelling evidence for causal involvement in developmental and psychiatric brain disorders. Most gene candidates are relatively uncharacterized compared to the decades of accumulated knowledge for some tumor suppressors and oncogenes. New exploratory research is needed to decipher the mechanistic details and organismal functions of those genes contributing to neurodevelopmental and neuropsychiatric disorders.

A prime candidate for focused attention is the understudied 25‐member *KCTD* gene family. Several human *KCTD* genes have emerged in association with neurodevelopmental, neuropsychiatric, and neurodegenerative disorders. Additional *KCTD* family members are associated with several types of cancer and other disorders, providing additional perspectives. While mutations in individual *KCTD* genes are found in a limited number of patients, collectively they provide a compelling basis to justify interrogation of their molecular and cellular functions to understand disease mechanisms. The biochemical and biological functions of KCTD proteins have not been deciphered, but progress is underway (Table 1). Some *KCTD* disease associations will need further validation, and likely many others are not yet identified. Deciphering the shared and distinct functions of multiple KCTD family members will provide a wealth of knowledge toward understanding neurodevelopmental, neuropsychiatric, and degenerative processes that were previously impermeable to interrogation.

**Table 1:** Disease associations, protein functions and structure determinations for all human KCTD family proteins and yeast Whi2.

Clade Figure 2	Protein	BTB structure	Binding partners	Biological functions	Disease relevance
E	**KCTD17**	closed pentamer (X‐ray^7^)	Cul3 2, 32 (5:5 SAXS 5, 7)	Promotes ciliogenesis by degrading trichoplein^32^, ^106^	Gen vars associated with dystonia^79^, ^83^
	KCTD5	closed pentamer (EM,^107^ X‐ray^3^)	Cul3^2^ (5:5 ITC^37^)	Inhibits GPCR signal, degrades Gβγ^18^; monoubiquitination of ΔNp63α^108^	Involved in sleep regulation^86^, ^109^
	KCTD2	ND	Clu3^29^	Degrades c‐Myc^29^ Regulates sleep^86^	Low in patient‐derived glioma stem cells 29 Gen vars assoc. with Alzheimer's risk (GWAS)^110^, ^111^
	KCTD9	Closed pentamer (X‐ray^2^)	Cul3 (5:5 cryo‐EM^2^)	ND	ND
D	SHKBP1	monomer (X‐ray^5^)	Cul3 (5:5 SAXS^5^) CIN85^112^ SETA^113^	Promotes EGFR pathway by disrupting c‐Cbl‐CIN85 complex^112^	Mutated in cervical cancer^114^ Mutated in leukemia^115^ Biomarker in small intestinal neuroendocrine tumors^116^
	**KCTD3**	ND	HCN3^91^	Up‐regulation of HCN3^91^	Biallelic mutations in epileptic encephalopathy^90^ Gen vars in intellectual disability/ seizures (WES)^88^, ^89^
C	KCTD10	tetramer (X‐ray^5^)	Cul3^31^, ^35^, ^117^ PCNA^6^ TNFAIP1^118^	Degrades RhoB^31^, ^35^ Promotes cilium, degrades CEP97^117^ DNA synthesis, cell proliferation^6^ Inhibits NF‐κB and AP‐1^118^	Tumor suppressor in gastrointestinal stromal tumor^119^
	TNFAIP1	ND	Cul3^33^, ^76^ RhoB^120^ PCNA^8^ KCTD10^118^	Degrades RhoA^33^, ^76^ Regulates apoptosis^120^ Inhibits NF‐κB and AP‐1 118	Aa a tumor suppressor in nonsmall cell lung cancer^121^ Poor prognosis if overexpressed in breast cancer^122^ Overexpressed in osteosarcoma^123^
	**KCTD13**	tetra‐ (X‐ray^5^) pentamer (EM^107^)	Cul3^11^, ^33^, ^76^ (5:5 SAXS^5^) SAXS^5^ PCNA^7^	Degrades RhoA^11^, ^33^, ^34^, ^76^	Copy‐number var associated with autism^11^, ^52^, ^76^ Mutations associated with schizophrenia^124^ Overexpression: microcephaly in zebrafish, mouse^34^
H	KCTD14	ND	ND	ND	ND
	**KCTD7**	ND	Cul3^13^, ^36^	Regulates neuronal autophagy,^13^ Gln transport SAT2,^23^ K^+^ conductance^20^	Bi‐allelic mutations cause severe early onset progressive disorder with epilepsy^13^, ^38^, ^39^, ^40^, ^41^, ^125^
B	KCTD6	pentamer (EM^107^)	Cul3^2^ (4:4 gel filtration^16^)	Suppresses Hh pathway by degrading HDAC^30^ and USP21^126^ Degrades small ankyrin‐1^127^	ND
	KCTD21	ND	Cul3^30^	Inhibits Hh by degrading HDAC^30^	Gen vars associated with autism (WES)^128^
	**KCTD11**	tetramer (gel filtration),^96^ pentamer (EM^107^)	Cul3 (4:4 gel filtration^16^, ^96^)	Inhibits mTORC1 activity^14^ Inhibits Hh pathway by degrading HDAC^12^	Deletion/ reduced expression in medulloblastoma^94^ Loss of heterozygosity in prostate adenocarcinoma^129^ Reduced expression in hepatocellular carcinoma^130^
Other	KCTD4	ND	ND	ND	ND
A	**KCTD15**	pentamer (EM^107^)	AP‐2α^10^	Inhibits neural crest formation by inhibiting AP‐2α^10^ & Wnt pathway^99^	Genetic variants associated with obesity^97^, ^98^
	KCTD1	closed/open pentamer (EM,^107^ X‐ray^2^)	AP‐2α transcription factor^9^	Inhibits transcription factor AP‐2α^9^ and Wnt signaling by degrading β‐catenin^131^	I27N mutation caused kidney dysfunction in mice^132^ Missense mutations associated with scalp‐ear‐nipple syndrome^133^
Other	KCTD19	ND	ND	ND	ND
F	**KCTD12**	pentamer (EM^107^; X‐ray^19^)	GABA_B2_ 17 Gβγ^19^ CDC25B^134^	Regulates GABA_B2 _receptor signaling^17^, ^19^, ^135^, ^136^ Suppresses Wnt‐Notch pathway^137^ Promotes G2/M transition^134^	Emotionality, neuronal excitability (mice)^65^ KCTD12 increases 5‐y survival in GI stromal tumor^138^ Increased KCTD12 in cervical and lung cancers^134^ Bipolar disorder (GWAS)^62^
	**KCTD16**	open pentamer (X‐ray^5^, ^19^)	GABA_B2_ 17 Gβγ^19^	Regulates GABA_B2_ receptor signaling^17^, ^19^, ^135^, ^136^	ND
	KCTD8	ND	GABA_B2_ 17	Regulates GABA_B2 _signaling^17^, ^135^, ^136^	ND
Other	KCNRG	ND	Kv channel^139^, ^140^	Suppresses K + channel activity^139^	Deleted in B‐cell chronic lymphocytic leukemia,^140^, ^141^, ^142^ prostate cancer^140^ and multiple myeloma^142^
Other	KCTD18	ND	ND	ND	Duplication of 2q33 in one patient with epilepsy, devel. delay, autistic behavior^143^ Haplotype associated with restless legs syndrome^144^
G	KCTD20	ND	ND	Activates Akt^145^, ^146^	Gen var associated with insulin resistance (GWAS)^147^
	BTBD10	ND	Akt1‐3^148^	Inhibits apoptosis, activates Akt^149^, ^150^	Sporadic amyotrophic lateral sclerosis^151^
*Sc*	**Whi2**	ND	Psr1^13^	Suppresses TORC1, promotes autophagy induction^13^	Plant pathogen CoWhi2 has suggested role in pathogenesis during infection^152^

Notes

5:5/4:4, pentameric or tetrameric symmetry when bound to binding partners; X‐ray/EM/gel‐filtration/SAXS‐small angle X‐ray scattering SAXS, structure determination methods.

Abbreviations: Gen vars, genetic variants associated with disease; ND, not determined; GWAS, genome‐wide association study; *Sc, Saccharomyces cerevisiae* (baker's yeast); WES, whole exome sequencing.

Bold type: KCTD proteins discussed in separate sections of this article.

**Figure 1:**
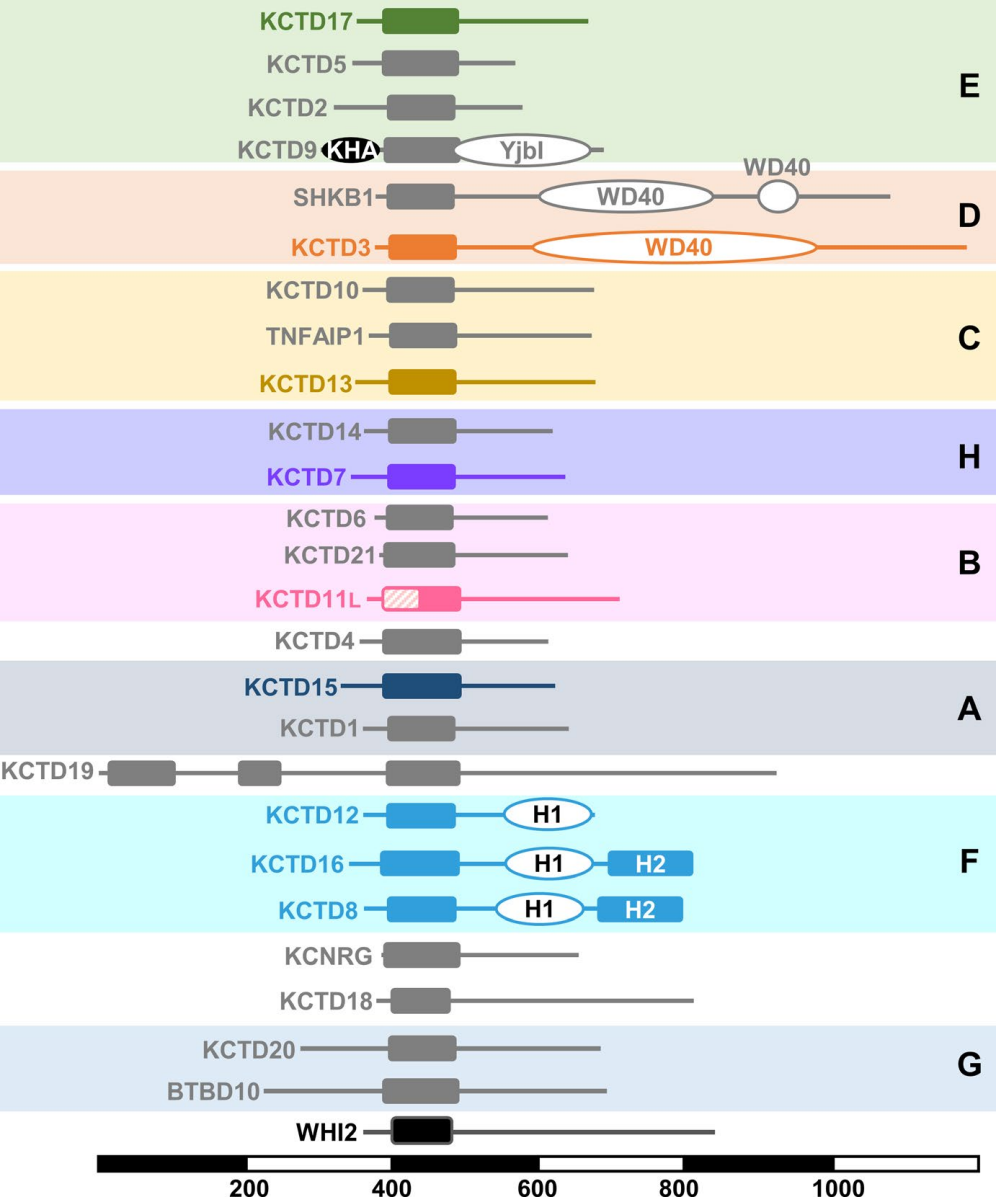
The diverse human KCTD protein family and yeast Whi2. Line diagrams of the 25 human KCTD family proteins and *Saccharomyces cerevisiae* Whi2 are drawn to scale, grouped in color‐coded clades (A‐H), ordered as in Figure 2, and aligned with respect to their BTB domain (solid rectangles). Additional protein domains with known or inferred structures (KHA, YjbI, WD40, H1) and similarity region H2 are also represented. KCTD11L starts at an AUU start codon adding 39 N‐terminal residues (hashed box) before the first in‐frame AUG translate start. Gray line diagrams indicate proteins not discussed in detail. Scale bar indicates protein length in amino acid residues

Human KCTD family proteins (KCTD1‐21, TNFAIP1, KCNRG, SHKBP1, and BTBD10) can localize in the cytoplasm or the nucleus, and range in size from 26‐kDa KCTD5 (234 amino acids) to 105‐kDa KCTD19 (926 amino acids). Mice encode an additional KCTD protein, Kctd12b (chromosome X), which is highly similar to mouse Kctd12 (chromosome 14). The distinguishing feature of KCTD proteins is a single N‐terminal BTB/POZ (bric‐a‐brac, tramtrak, and broad complex/poxvirus zinc finger) domain, the exception being KCTD19 with three separate BTBs that may reflect tandem gene amplification (Figure 1).^1^ BLAST searches readily reveal that the BTB domains of KCTD family proteins are most similar in amino acid sequence to the T1/BTB domains that mediate tetramerization of voltage‐gated potassium channel subunits to form functional channels.^1^, ^2^ This sequence similarity to Kv channels explains how KCTDs acquired their official name (potassium channel tetramerization domain). However, KCTD family proteins lack predicted transmembrane domains.^3^

An unusual feature of this gene family is their diversity outside the BTB domain. Except within subgroups of closely related family members, KCTD proteins lack obvious sequence similarity in their highly variable C‐terminal regions.^1^ This feature is consistent with their proposed roles as adaptor molecules that use their C‐termini to bind and recruit diverse cellular proteins destined for degradation. In this model, KCTDs are responsible for selecting protein substrates for ubiquitination by cullin‐RING ubiquitin ligases (CRLs) that bind to the BTB domains of KCTD proteins.^2^, ^4^, ^5^ Thus, disrupted proteostasis required for the delicate balance between protein function versus degradation could potentially underlie neurological disorders now associated with the brain‐enriched proteins KCTD3, KCTD7, KCTD13, and KCTD17. This proposed adaptor function for KCTD proteins could potentially underlie the diverse biological processes reported for KCTD proteins, including DNA replication (KCTD10 and TNFAIP1),^6^, ^7^, ^8^ transcription inhibition (KCTD1 and KCTD15),^9^, ^10^ regulation of Rho GTPases in brain development (KCTD13),^11^ suppression of hedgehog signaling (KCTD11),^12^ autophagy induction and amino acid signaling to mTORC1 (KCTD11),^13^, ^14^, ^15^ and more (Table 1). However, other KCTDs appear to lack the ability to bind cullin‐3, implying distinct biochemical mechanisms.^16^ For example, several KCTD family proteins (e.g., KCTD12 and KCTD13) may alter neuronal activity and other signaling pathways by regulating diverse types of GTPases 11, 17, 18, 19. Here, we convey our current understanding of KCTD protein structure and function and how this may relate to disease mechanisms, focusing on a subset of KCTD family members implicated in disorders originating from the neural crest.

## 2 STRUCTURE AND FUNCTION OF KCTD FAMILY PROTEINS

**Figure 2:**
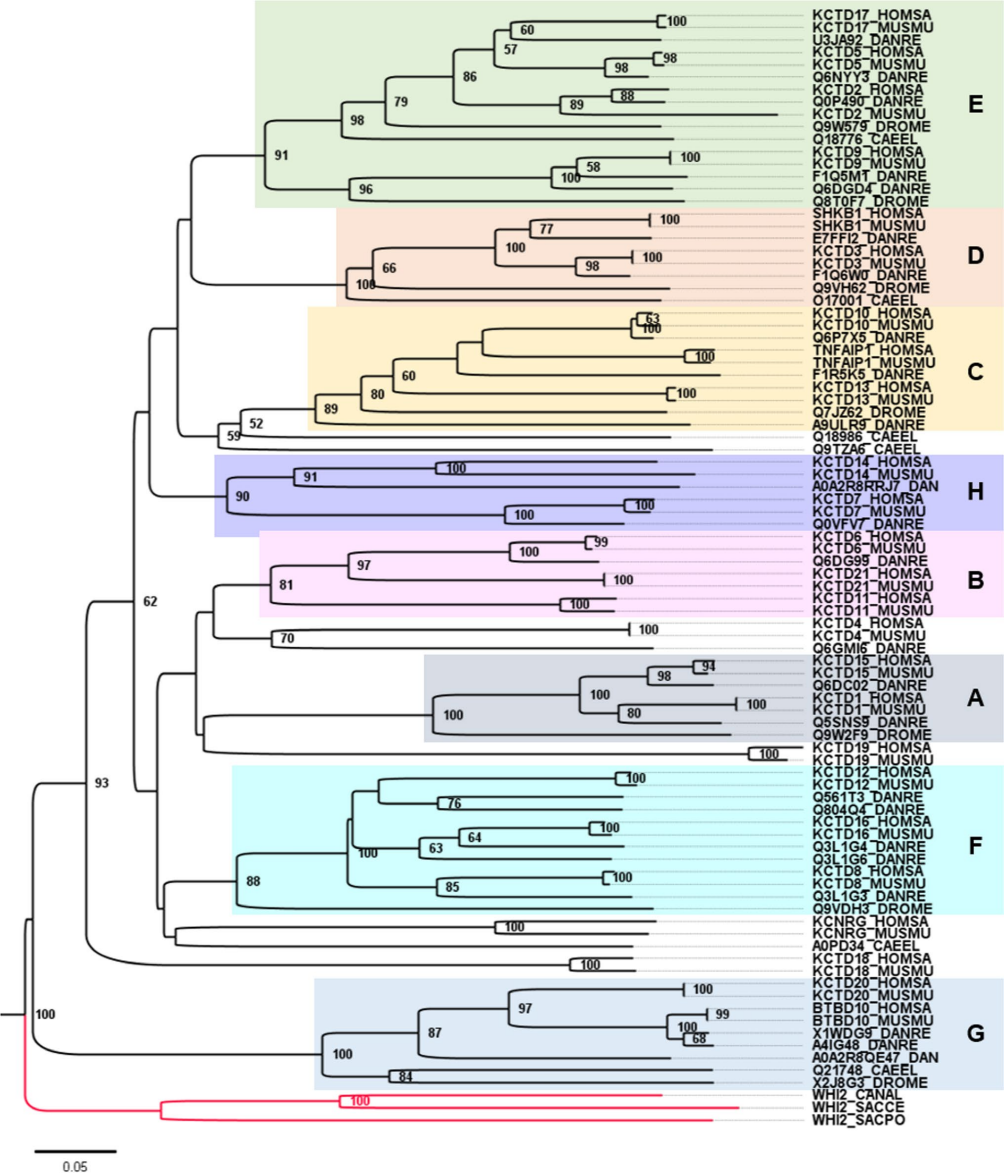
Phylogenetic tree of isolated BTB domains from KCTD family homologs. Amino acid sequences of KCTD family proteins from human (*Homo sapiens*, HOMSA), mouse (*Mus musculus,* MUSMU), zebrafish (*Danio rerio*, DANRE), *Drosophila melanogaster* (DROME), *Caenorhabditis elegans* (CAEEL), and three yeast species (*Saccharomyces cerevisiae*, SACCE; *Schizosaccharomyces pombe*, SACPO; *Candida albicans*, CANAL) were collected from UniProt (release 2019_02) or after searches using the DELTA‐BLAST algorithm on the NCBI website. Sequences were aligned using MAFFT (version 7), and a neighbor‐joining (NJ) analysis was performed with 1000 bootstrap replicates. Bootstrap support values above 50 are shown at each node. The tree was rooted using Whi2p from *S pombe*. Yeast sequences were represented as an outgroup (red branches). The arbitrary cluster designations for groups A‐G were assigned to match those reported by Skoblov *et al*.^1^ The new H group is deduced from this analysis. Compared to Skoblov *et al*,^1^ we found that KCTD9 segregates within group E. Amino acid sequences (Table S1) and alignment results (Table S2) for this analysis are found in Supporting information

KCTD family members likely represent paralogs that arose by gene duplication from a common ancestral gene followed by divergence, which led to diversification of the current KCTD protein family in the animal kingdom (Figure 2). Solved structures are available for the N‐terminal BTB domain of several KCTD family proteins primarily revealing pentameric homo‐oligomers. This fivefold symmetry appears to extend through the C‐terminus.^1^, ^2^, ^3^, ^19^ The BTB domains of KCTD proteins also mediate other protein‐protein interactions, leading to three main hypotheses for general KCTD mechanisms. The first of these is not favored currently. Reasoning that KCTD‐BTB domains might directly bind to their closest cousins, the T1/BTB (tetramerization) domains of voltage‐gated potassium channels, KCTD proteins could potentially regulate channel assembly or activity. KCTD5 was tested for the ability to bind or affect the functions of Kv1.2, Kv2.1, Kv3.4, and Kv4.2 channels but without success.^3^ In a second model, several KCTD family members are reported to indirectly influence channel activity by mechanisms not yet delineated.^20^, ^21^, ^22^, ^23^ However, recent advancements in this direction stem for the finding that the BTB domain of a different subset of KCTDs (clade F) interacts with the cytoplasmic tail of membrane‐embedded GABA_B_ neurotransmitter receptors allowing the KCTD C‐terminus to transmit a signal that modulates channels in close proximity.^19^ The third proposed biochemical mechanisms for KCTD family proteins potentially apply more broadly to the KCTD protein family. The BTB domain of many KCTD family proteins are reported to bind to the cullin‐3 ubiquitin ligase, potentially serving as adaptor molecules to recruit substrates for ubiquitination.^5^ However, exactly how the KCTD BTB domain of KCTD proteins would fit onto cullin‐3 is a matter of speculation. Furthermore, little is known about the structure or function of most KCTD C‐termini.

KCTD family proteins were previously classified into seven phylogenetic clades based on the amino acid sequences of the BTB regions alone or of the full‐length proteins.^1^ Our analysis based on the minimal BTB domains is in agreement with the previous study and suggests the existence of an additional 8th clade that we termed H comprised of KCTD7 and KCTD14 (Figures 1, 2). In addition, based on our analysis, we propose to include the BTB of KCTD9 in the E group. Like the related tetrameric T1/BTB domains of voltage‐gated potassium (Kv) channels, the BTB domains of KCTD10 and KCTD13 are capable of forming tetramers. However, the BTB domains of most KCTD proteins form pentamers (KCTD1, −5, −6, −9, −11, −12, −15, −16, −17) based on crystal structures, cryo‐EM, or other methods (Table 1). The exception is the available structure of SHKBP1‐BTB, which is a monomer.^5^ The only available full‐length KCTD protein structure reveals a fivefold symmetry extending through the C‐terminus of KCTD5,^3^ consistent with the pentameric structure of the C‐terminal H1 domain of KCTD12.^19^

**Figure 3:**
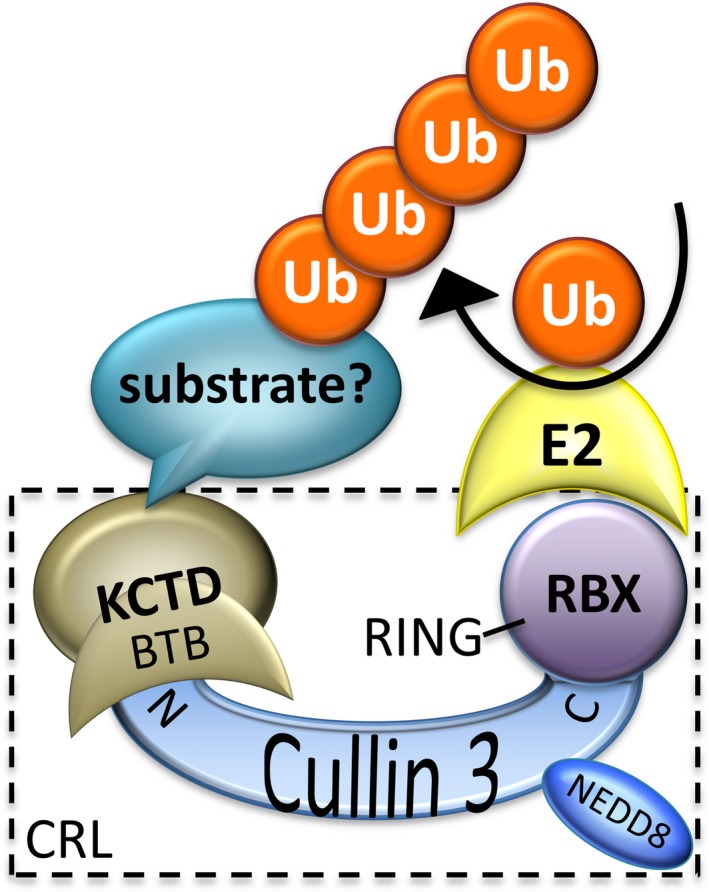
Proposed role for a subset of KCTD family proteins as adaptors for cullin‐3 ubiquitin ligase complexes (CRLs)

BTB domains are found in other well‐known proteins, such as Skip1, an adaptor for cullin‐1 ubiquitin ligase complexes, and KEAP1, which regulates localization of NRF2 in a redox‐responsive manner.^24^ The identification of BTB domains from other protein families as cullin‐binding partners by mass spectrometry, including several KCTD family members,^25^, ^26^, ^27^ fuels the search for biological roles for KCTD family proteins as exchangeable adaptors of cullin‐3‐RING E3 ubiquitin ligase complexes (CRLs). In this model, cullin‐3 interacts with the BTB domains of exchangeable adaptor proteins that serve to recruit different protein substrates for ubiquitination by the RBX‐RING protein bound to the C‐terminus of cullin‐3 (Figure 3).^27^, ^28^ Thus, KCTD family proteins could function like a multihead screwdriver to recruit different cellular proteins for selective degradation by the proteasome or the lysosome to fine‐tune many cellular processes.^27^

Several KCTD family proteins (KCTD2, −5, −6, −10, −11, −13, −17, −21, TNFAIP1) have been reported to interact with cullin‐3 and to mediate ubiquitination and degradation of specific target proteins.^11^, ^12^, ^18^, ^29^, ^30^, ^31^, ^32^, ^33^ Cullin‐3 binding to KCTD13, TNFAIP1, and KCTD10 may regulate actin organization and other cell functions by degrading Rho GTPases RhoA or RhoB.^31^, ^33^, ^34^, ^35^ KCTD6, KCTD11, and KCTD21 can each assemble with cullin‐3 and are reported to suppress hedgehog signaling by degrading Gli deacetylase HDAC1.^12^, ^30^ KCTD7, KCTD9, and SHKBP1 are capable of binding cullin‐3, but their substrates have not yet been identified.^2^, ^5^, ^36^ Intriguingly, KCTD5, −9, −13, −17, and SHKBP1 can form 5:5 heterodecameric complexes with cullin‐3 based on biochemical experiments, even though their purified BTB domains may adopt geometries of tetramers or monomers.^2^, ^5^, ^37^ This suggests that cullin‐3 may drive assembly of KCTD protein structures. Validation of this adaptor function by in vitro reconstitution of KCTD‐cullin3‐RBX‐E2 ubiquitination reactions is challenged by the apparent need to identify and include the specific target substrate in these reactions. Detailed biochemistry and structure determinations are also needed to confirm the biological evidence that KCTDs function as cullin‐3 adaptors. Additional binding partners of KCTD proteins also have been identified. KCTD1 and KCTD15 were reported to bind and inhibit the activity of transcription factor AP‐2α.^9^, ^10^ KCTD10, KCTD13, and TNFAIP1 were reported to regulate DNA replication by interacting with PCNA (proliferating cell nuclear antigen).^6^, ^8^ Whether these KCTD functions involve cullin‐3 or unrelated mechanisms is not yet established.

## 3 *KCTD* GENES ASSOCIATED WITH NEURODEVELOPMENTAL AND NEUROPSYCHIATRIC DISORDERS

### 3.1 KCTD7 mutations cause a severe neurodevelopmental disorder

Our recent genetic analysis confirms that mutations in *KCTD7* cause a rare early‐onset, autosomal recessive disorder (progressive myoclonic epilepsy/PME3, also called EPM3).^13^ Over 50 patients with over 40 unique variants in *KCTD7* have been identified to date, though many more likely remain unidentified.^13^, ^38^, ^39^, ^40^, ^41^, ^42^, ^43^, ^44^, ^45^ In all cases, patients have homozygous or compound heterozygous mutations (missense, stop‐gain, frameshifts, or large deletions), while all heterozygous family members are unaffected.^13^ Thus far, the highest density of patient variants occurs within the N‐terminal BTB domain. A cluster of mutations also occurs in the last 30 residues and in an ~100‐residue middle region, both of unknown function.^13^ Patients appear to develop normally and achieve early childhood milestones. However, between 10 and 20 months of age these children develop refractory myoclonic seizures, movement disorders, and/or developmental delays.^13^ This younger age of onset with *KCTD7* mutations (average 16 months) distinguishes these patients from other types of progressive myoclonic epilepsies (PME). All patients subsequently progress, exhibiting severe cognitive decline, motor deficits, and seizures.^13^ Most patients become nonverbal and wheelchair‐bound within 2 years of diagnosis, but the few ambulatory patients now in their teens and early twenties have diagnoses of autism or schizophrenia. This disorder is also designated as neuronal ceroid lipofuscinosis type 14 (CLN14), primarily on the basis of two patients with subcellular inclusions and lysosome storage material.^36^, ^45^ However, most studies concluded that the subcellular pathologies of KCTD7 patients are distinct from previously described CLN pathologies and other lysosomal storage disorders.^13^, ^41^

Persistent difficulties with diagnosing this disorder have been attributed to earlier onset ages than expected for PME disorders, negative biopsy tests for CLN‐related pathologies, negative brain MRI findings (with a few exceptions), and the fact that *KCTD7* was not confirmed as a disease gene until relatively recently. These challenges have been partially overcome by including *KCTD7* on diagnostic sequencing panels for epilepsy. However, at least 25% of patients develop movement disorders (ataxia, tremors, dyskinesia, choreoathetosis, dystonia) or regression of milestones before the onset of seizures, though all eventually develop myoclonic epilepsy. Electrical activity in the brain detected by EEG tests is generally positive, and brain biopsies are predicted to be diagnostic based on the prevalence of lipofuscin/lysosome‐like structures in patient brain.^13^ One patient underwent callosotomy with reported benefit.^13^ A number of patients with heterozygous *KCTD7* mutations and some overlapping neurological symptoms have also been identified, although any causal role for *KCTD7* in these cases is unknown.^13^ Undiagnosed bi‐allelic *KCTD7* gene mutations have contributed to the misassignment of disease symptoms to unrelated events such as vaccinations routinely administered around the expected age of disease onset. In addition, the gene name for *KCTDs* has evoked assumptions that patients could be treated for a channelopathy,^46^ though currently available evidence does not justify this therapeutic approach.

Although the molecular mechanisms underlying disease in patients with *KCTD7* mutations are not known, research efforts have begun to dissect some biological functions. KCTD7 protein expression was reported in hippocampal neurons and Purkinje cells of mouse brain.^20^, ^41^ Expressed wild‐type KCTD7 protein in cultured mouse neurons or *Xenopus* oocytes was reported to hyperpolarize the resting cell membrane potential, and some patient mutations inhibited the K^+^ flux observed with wild‐type KCTD7.^20^, ^23^ How KCTD7 might influence potassium currents is not known but may be indirect as compelling evidence of a direct interaction with K^+^ channels is currently lacking. Cerebrospinal fluid from some patients was reported to have higher levels of glutamine and lower levels of glutamate, which was suggested to result from impaired regulation of the neuronal glutamine transporter SAT2 by mutant KCTD7.^23^

Our biochemical studies indicate that KCTD7 protein interacts with cullin‐3,^13^, ^20^ raising the possibility that KCTD7 may serve as an adaptor for the cullin‐3 E3 ubiquitin ligase to mediate protein degradation of targeted substrates. However, no KCTD7‐recruited substrate proteins have been identified. One study implied that the C‐terminus of KCTD7 may be involved in binding cullin‐3.^36^ More recent evidence indicates that the BTB‐containing N‐terminus of KCTD7 is required and sufficient for cullin‐3 interactions based on co‐immunoprecipitation assays and subcellular localization of expressed proteins.^13^ Furthermore, this interaction with cullin‐3 is partially impaired by BTB domain mutations found in patients (R70W, R84Q, L108M).^13^ Thus, a role for KCTD7 in proteostasis could conceivably contribute to progressive disease by causing the accumulation of undegraded proteins, correlating with the prevalence of abnormal lysosome‐like structures observed by electron microscopy in neurons of a patient brain biopsy.^13^ Consistent with these findings, electron microscopy analysis of low‐passage skin fibroblasts derived from two additional KCTD7 patients exhibits abnormal mitochondrial cristae morphologies, lipid droplet accumulation around mitochondria, and phagolysosomes containing partially degraded material, features that were absent from matched control cells.^13^

One potential mechanism to explain these observations arose from yeast genetic studies. Fungal Whi2 protein sequences and metazoan KCTD family sequences share a homologous BTB domain (IPR011333) with significant sequence similarity (Figures 1, 2).^47^ It is not known whether both yeast and mammalian KCTD proteins descended from a common ancestral gene or whether they evolved as a result of BTB domain insertion into unrelated ancestral genes (therefore, they share sequence homology but are not referred to as homologs). The yeast *WHI2* gene from *Saccharomyces cerevisiae* was originally discovered when a spontaneous inactivating mutation in *WHI2* was identified as the cause for a cell growth phenotype.^48^, ^49^ Yeast *WHI2* was later rediscovered for similar reasons, because spontaneous *WHI2* mutations caused cells to continue growing inappropriately after switching cells to medium with low levels of amino acids.^50^ This is because Whi2 is required to suppress TORC1 kinase, the master regulator of cellular responses to nutrient status.^14^, ^15^, ^51^ Interestingly, knockdown of *Kctd13* in neuro2A cells was reported to increase cell proliferation.^52^ Whether KCTD13 or KCTD7 regulates TORC1, or whether this involves cullin‐3‐dependent protein degradation is not known. However, given that TORC1 is well known to actively suppress autophagy in yeast and mammals, it is not surprising that *whi2* ‐mutant yeast, which have sustained TORC1 activity in low amino acid conditions, fail to induce autophagy.^13^ Interestingly, *KCTD7* patient fibroblasts were found to have defective autophagy induction when starved.^13^ Consistent with a role for BTB‐containing, cullin‐interacting proteins in autophagy regulation, the BTB‐kelch‐repeat protein KLHL20 regulates autophagy by functioning as a cullin‐3 adaptor to degrade the mTORC1‐inhibited ULK1 protein kinase and the lipid kinase VPS34, both important for early steps of autophagosome formation.^53^ Similarly, the F‐box protein and associated BTB/POZ protein Skp1 can mediate cullin‐1‐dependent degradation of VPS34 to regulate autophagy.^54^

**Figure 4:**
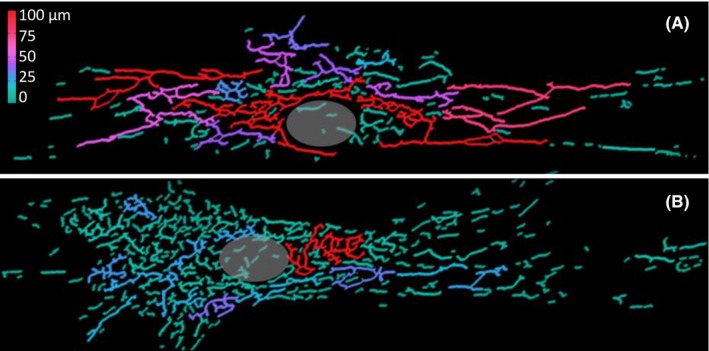
Altered mitochondrial morphology in *KCTD7* mutant patient fibroblasts. Primary passage‐matched human fibroblasts from (A) an age‐matched control and (B) a patient with compound heterozygous R84W/D106fs mutations in *KCTD7* were confirmed by Sanger sequencing and qRT‐PCR analysis as described.^13^ To visualize mitochondrial organelles, cells grown on round 12‐mm‐diameter glass coverslips (FisherBrand) were fixed (10 min in cold 4% paraformaldehyde), permeabilized (5 min with 0.2% Triton X‐100) and immunostained 1 h with anti‐Tom20 antibody and Alexa Fluor® secondary antibodies (Santa Cruz), mounted in Prolong Gold, and 0.5 μmol/L Z‐stack images were captured on a Nikon 90i at 40x or 60x magnification using Volocity software for deconvolution. (For quantification, mitochondria in some experiments were labeled instead with 100 nmol/L Mitotracker Red for 15 min prior to fixation.) Double‐blinded images were converted to 8‐bit grayscale, binarized and skeletonized using a custom ImageJ plug‐in, and mitochondrial structure parameters (including length, size, branching, degree of clustering, circularity) were quantified using “Analyze Skeleton 2D/3D” ImageJ plug‐in for 2‐3 independent experiments. The total mitochondrial network per cells was significantly reduced in long‐branch frequency in *KCTD7* mutant fibroblast compared to control fibroblast. Individual mitochondrial subnetworks (skeletons) are rainbow colored according to total length (red longest, blue shortest). Position of the nucleus in each cell is marked by a gray circle

Interestingly, a spontaneous *whi2* mutation in yeast partially rescues defective mitochondrial respiratory function (petite phenotype) caused by loss of the mitochondrial fission factor Fis1, also conserved in humans.^47^, ^50^ By promoting mitochondrial organelle fission, Fis1 is thought to promote turnover of mitochondria by generating small organelles that can be engulfed by autophagosomes.^55^, ^56^ Perhaps sustained TORC1 activity in *fis1whi2* double mutants helps compensate for mitochondrial insufficiency without Fis1, explaining why most *FIS1*‐deletion strains develop a secondary *WHI2* mutation.^50^ While the role of Whi2 versus Fis1 in mitochondrial turnover via mitophagy is debated,^57^, ^58^ the profound defect in autophagy observed in knockouts lacking Whi2 (yeast KCTD) provided the first clue about the function of human KCTD7 in autophagy. This model is consistent with the prevalence of mitochondria containing defective cristae membrane structures 13 and the altered branching patterns of mitochondrial organelles observed in KCTD7 patient fibroblasts (Figure 4). In the future, animal models will likely be needed to understand the physiological and pathological consequences of *Kctd7* deficiency before grasping the organismal and behavioral consequences relevant to human disease mechanisms.

### 3.2 KCTD8, KCTD12, and KCTD16 in neurotransmitter receptor signaling

Mouse Kctd8, Kctd12, Kctd12b (not found in humans), and Kctd16 belong to clade F of the KCTD protein family (Figure 2, Table 1)^1^ and are considered auxiliary subunits of the inhibitory neurotransmitter receptor complex GABA_B1/2_ (G‐protein‐coupled receptor/GPCR 3 family) present on both inhibitory and excitatory neurons.^17^, ^59^ Supported by studies in GABA_B_‐deficient mice, GABA_B_ receptor aberrations are implicated in neurodegenerative and neuropsychiatric disorders, including seizure disorders, depression, schizophrenia, addiction, and several neurodevelopmental disorders.^59^, ^60^, ^61^ Therefore, disruption of the auxiliary subunits KCTD8, KCTD12, and KCTD16 may cause related conditions. A mutation in the promoter region of human *KCTD12* was reported to contribute to bipolar I disorder.^62^ Similarly, elevated protein levels of human KCTD12 were associated with depression^63^ and schizophrenia.^64^ Consistent with these findings, *kctd12* ‐knockout mice exhibit related phenotypes including altered emotional behaviors and increased neuronal excitability,^65^ supporting a potential role for *KCTD12* in neuropsychiatric disorders. *KCTD12* has also been implicated in several cancers not discussed here.

Insights into the molecular mechanisms involved are at the forefront of understanding KCTD family protein functions, and recent structure determinations further advance the field overall. The homologous GABA_B1_ and GABA_B2_ receptors (*GABBR1* and *GABBR2*) function as heterodimers. GABA_B1_ binds the inhibitory neurotransmitter gamma‐aminobutyric acid (GABA), and the GABA_B2_ subunit interacts with G‐proteins for signaling. GABA_B1/2_ receptors modulate synaptic transmission by indirectly regulating specific Ca^2+^ and K^+^ channels through trimeric G‐proteins.^66^ KCTD8, KCTD12, and KCTD16 can increase the activation rate of GABA_B_ responses, and KCTD12 can cause fast desensitization of GABA_B_ receptor responses.^17^, ^21^, ^67^ A new crystal structure containing the C‐terminus of GABA_B2_ (amino acid residues 876‐913) reveals how the pentameric BTB domain of KCTD16 enwraps the cytoplasmic tail of the neurotransmitter receptor GABA_B2_.^19^ The interaction with KCTD16 and also with KCTD12 is abolished by the BTB mutation Phe80Ala in KCTD16 and Phe87Ala in KCTD12, further validating the crystal structure.^19^

The same study also connects KCTD proteins with trimeric G‐protein complexes, providing a model for how clade F KCTDs may transmit a signal to regulate potassium flux across the cell membrane. The conserved H1 and H2 homology regions were previously recognized in the C‐terminus of clade F proteins, except H2 is not present in the shorter KCTD12 C‐terminus (Figure 1).^21^ The KCTD12 H1 region was previously shown to be responsible for desensitization of GABA_B_ receptor responses, whereas H2 domains of KCTD8 and KCTD16 have auto‐inhibitory effects on their H1 region.^21^ A new crystal structure of KCTD12 H1 bound to Gβ_1_γ_2_ reveals an H1 pentamer surrounded by five Gβ_1_γ_2_ dimers.^19^ Taking the evidence together, the proposed model is that a KCTD12 pentamer dangles from the extended cytoplasmic tail of GABA_B2_, which is anchored in the cell membrane with GABA_B1_.^19^ Upon GABA_B1_ receptor stimulation, KCTD12 expels Gα from the inhibited trimeric G‐protein complex Gαβ_1_γ_2_, and membrane‐associated Gβ_1_γ_2_ can rapidly activate the associated GIRK (G‐protein‐coupled inwardly rectifying K^+^ channel). Then, rapid deactivation/desensitization of GIRK channels would subsequently occur when KCTD12 H1 sequesters Gβγ away from these channels.^19^ A role for KCTD12 in GABA_B_‐Gβγ signaling to regulate potassium channel activity is not mutually exclusive with a role as a cullin‐3 adaptor (e.g., to degrade Gα), except that the α2β3 loop of KCTD12 is predicted to interfere with cullin‐3 interactions.^16^ Therefore, any congruency between the cullin‐binding KCTDs and GTPase signaling KCTDs is currently unresolved but may represent independent functions of the same or different KCTD family proteins. However, it is tempting to consider that these functions could be present in the same KCTD protein to coordinate cellular functions.

### 3.3 KCTD13 association with autism and schizophrenia

Recent genetic studies have revealed copy‐number variations (CNV) in many genes in association with developmental brain disorders, intellectual disability, epilepsy, autism spectrum disorder, and schizophrenia.^68^ *KCTD13* (also known as *BACURD1* or *POLDIP1*) is located in the 16p11.2 locus, which is known to contribute to risk of multiple neuropsychiatric disorders. Deletions of 16p11.2 are associated with epilepsy, autism, and autism spectrum disorder (ASD),^69^ while 16p11.2 duplications are associated with autism and schizophrenia.^70^ Interestingly, dosage effects of 16p11.2 appear to affect head size in humans, with deletions observed in macrocephaly and duplication observed in microcephaly.^70^

Zebrafish and mouse model systems have helped to overcome the major challenge of dissecting the individual contributions to disease of the many genes present 16p11.2 duplications/deletions. Zebrafish have been useful in other studies to investigate human dosage‐sensitive genes and can reflect anatomical phenotypes observed in early human development.^71^ Therefore, zebrafish were used to dissect human 16p11.2, which encompasses 29 genes that when deleted in humans can confer susceptibility to neurocognitive defects.^52^, ^69^ Results from overexpression of each of these 29 genes individually in zebrafish embryos identified a single gene, *KCTD13*, capable of inducing microcephaly, a phenotype of patients with 16p11.2 duplication.^52^ Conversely, transient suppression of the orthologous Zebrafish *kctd13* locus resulted in the reciprocal macrocephaly phenotype.^52^, ^72^, ^73^ The importance of *Kctd13* for cellular proliferation was confirmed in developing mouse brains. In contrast to these studies, others failed to detect increased brain size or increased neurogenesis in mice or zebrafish when the entire *Kctd13* locus was deleted in zebrafish or mice.^11^ The discrepancy between both lines of data may be due to different compensation mechanisms between knockdown approaches and genetic deletion, possible phenotypic differences between *Kctd13* knockdown in only a subset of neural progenitors versus complete genetic deletion of the *Kctd13 locus*, or alternatively, contributions from other genes located in 16p11.2.

Other studies demonstrate that in mouse models, *Kctd13* epistatically affects anatomical phenotypes in combination with *Mvp* (major vault protein) or *Lat (linker for activation of T cells)*, genes, which are also located in 16p11.2 loci^52^, ^74^. However, recent studies reported no robust abnormalities in brain structure of mice with genetic ablation of *Kctd13*, and instead observed sex‐specific differences in brain volume of double heterozygous mice lacking one copy of *Kctd13* and one copy of either *Lat* or *Mvp*, also located in 16p11.2.^75^ These results suggest that altered dosage of *Kctd13*, and *Mvp* or *Lat* may have epistatic effects on brain size.

KCTD13 has been reported to function as a cullin‐3 adaptor for ubiquitination and degradation of RhoA, a small GTPase protein that is a key regulator of actin cytoskeleton and plays critical roles in neuronal development and synaptic function.^33^, ^76^ Consistently, genetic deletion of the entire *Kctd13* gene has resulted in increased RhoA expression, the loss of dendritic spines and reduced synaptic activity in the CA1 region of the hippocampus.^11^, ^76^ Reduced synaptic transmission is normalized by pharmacological inhibition of RhoA. These results suggest that KCTD13 may recruit RhoA for modulating its turnover via the cullin‐3 ubiquitin ligase, thereby regulating synaptic function, consistent with spatiotemporal network analysis of brain subregions.^34^ This *Kctd13*‐knockout mouse (entire *Kctd13* gene deleted) lacked detectable memory deficits.^11^ However, an independently constructed *Kctd13*‐deficient mouse with an out‐of‐frame exon 2 deletion (expected to fully ablate *Kctd13*) exhibited deficits in short‐term recognition memory, but lacked detectable changes in expression levels of RhoA, the candidate KCTD13‐cullin target substrate.^75^ However, RNA‐seq analyses of gene expression profiles from the cortex and hippocampus of *Kctd13* ‐deficient (exon 2‐deleted) mice revealed altered signaling pathways critical for neurodevelopment, including synaptic formation, and both knockout mouse lines exhibited reduced spine density in the hippocampus.^11^, ^75^ Thus, further studies are required to understand the mechanistic complexities by which Kctd13 copy number modulates brain development beyond RhoA signaling. It would also be of great interest to investigate how multiple genes in 16p11.2 loci interplay to regulate brain development and contribute to neurodevelopmental abnormalities and psychiatric disorders such as schizophrenia and autism.

### 3.4 KCTD17 in myoclonus‐dystonia

Myoclonus‐dystonia syndrome (MDS) is a rare movement disorder characterized by nonepileptic spontaneous muscle contractions and dystonia.^77^ Approximately 25‐50% of myoclonus‐dystonia cases are caused by autosomal dominant mutations in the *SGCE* gene, coding for ε‐sarcoglycan.^78^ Thus, there are additional genetic variants responsible for this disease, and several candidate genes have been identified, some of which have been confirmed.^79^, ^80^ *KCTD17* variant c.434G > A, p.Arg145His was identified by combining genome‐wide linkage analysis and whole‐exome sequencing of a large British pedigree and of a second German family with autosomal dominant myoclonus‐dystonia but lacking *SGCE* gene mutations.^79^ Additional tests confirmed the lack of a common ancestor between these two families. *KCTD17* (c.434G > A, p.Arg145His) was the only segregating variant among seven candidates from affected myoclonus‐dystonia patients in the British family.^79^ Very recently, two additional *KCTD17* mutations affecting the same splice acceptor site (c.508‐2A > T and c.508‐1G > T) were identified in two independent studies.^81^, ^82^ It has been pointed out that the clinical features of the *KCTD17* patients are phenotypically distinguishable from MDS due to *SGCE* mutations.^80^ However, the evidence is reasonably compelling that *KCTD17* mutations are responsible for a subset of myoclonus‐dystonia. Although autosomal dominant *KCTD17* mutations cause less severe disease than bi‐allelic *KCTD7* mutations discussed in section 3.1, both *KCTD7* and *KCTD17* disorders have some overlapping clinical features including difficulty swallowing, impaired verbal skills, cognitive impairment, difficulties with fine motor skills, and their disease is progressive, unlike SGCE mutations.

The biological functions of KCTD17 are not yet clear. *KCTD17* mRNA was shown to be broadly expressed across the brain but particularly in the putamen, consistent with dystonia being caused by dysfunction of basal ganglia circuits.^79^ Fibroblasts derived from a *KCTD17* patient (p.Arg145His) exhibit defective ER calcium signaling, which is suggested to underlie myoclonus‐dystonia linked to mutations in other genes (e.g.,* HPCA*, *CACNA1A*, *ANO3*).^79^, ^83^

KCTD17 has also been reported to function as an adaptor of the cullin‐3 ubiquitin ligase to mediate ubiquitination and degradation of trichoplein, which is a negative regulator of ciliogenesis.^32^ Both neurons and astrocytes contain a primary cilium, and ciliogenesis has been reported to play an important role in brain development.^84^ This raises the possibility that defective ciliogenesis caused by *KCTD17* mutations contributes to the pathology of myoclonus‐dystonia.

The BTB domain of mammalian KCTD17 is most similar in sequence to KCTD2, KCTD5, and KCTD9, which together constitute clade E (Figure 2, Table 1).^1^ Mammalian KCTD2, KCTD5, and KCTD17 are homologs of *Drosophila* Insomniac protein (Inc), a regulator of sleep homeostasis and synaptic function in flies.^85^ Insomniac was reported to be a substrate adaptor of *Drosophila* cullin‐3 and may regulate turnover of yet unknown neuronal targets to regulate sleep and synaptic functions.^85^ Both insomniac and its mammalian homologs are expressed in the nervous system and localize to synapses.^86^ Mouse KCTD2, KCTD5, and KCTD17 can each heteromultimerize with *Drosophila* Insomniac and also bind to *Drosophila* cullin‐3 in vitro*,* suggesting conserved functions.^86^ Although only mouse KCTD2 and KCTD5, but not KCTD17, were able to rescue the sleep phenotype in flies lacking *Insomniac*, the inability of KCTD17 to restore sleep in *insomniac* mutant flies was suggested to be due to its low expression in transgenic flies.^86^ *Drosophila* Insomniac and cullin‐3 also regulate dopaminergic signaling.^85^ Dysfunction of dopaminergic pathways has been associated with myoclonus‐dystonia.^87^ Currently, the molecular links between KCTD17‐cullin3‐dependent protein degradation, synaptic function, dopaminergic signaling, and pathology of myoclonus‐dystonia remain unclear.

### 3.5 KCTD3 in neurocognitive disease

KCTD3, also known as NY‐REN‐45, has been identified in several genome‐wide screens for disease variants. A bi‐allelic frameshift mutation in *KCTD3* (c.1036_1073del, p.P346Tfs*4) was first identified in one family by whole exon sequencing of 143 multiplex families with neurocognitive disorders.^88^ This same *KCTD3* mutation was later reported in a 2.5‐year‐old patient.^89^ Homozygous *KCTD3* mutations were identified in three additional families, one harboring the same frameshift mutation (c.1036_1073del, p.P346Tfs*4), and the other two harboring a missense mutation (c.166C > T, p.Arg56*).^90^ *KCTD3* patients exhibit global developmental delay, seizures, and cerebellar hypoplasia.^88^, ^90^

The biological function of KCTD3 protein has not been investigated in‐depth, but one study provides some evidence that links KCTD3 with the nervous system. Mouse Kctd3 was identified as a binding partner of Hcn3 (hyperpolarization‐activated cyclic nucleotide‐gated channel) in a yeast two‐hybrid screen.^91^ Immunoprecipitation from mouse brain lysates suggests that Kctd3 specifically binds to Hcn3, but not to the other Hcn channels (Hcn1, Hcn2, and Hcn4).^91^ Immunostaining confirmed that Kctd3 and Hcn3 colocalize in several brain regions including hypothalamus, midbrain and cerebellum. Kctd3 increases Hcn3 current density by promoting trafficking of Hcn3 protein to the cell membrane.^91^ Human HCN channels are widely expressed in the brain and are reported to control cellular excitability and synaptic transmission.^92^, ^93^ Whether KCTD3 also regulates these neuronal functions is not yet known.

## 4 RELEVANCE OF OTHER KCTD FAMILY MEMBERS TO THE NERVOUS SYSTEM

### 4.1 KCTD11 in cancer

KCTD11 has been implicated as a tumor suppressor in several cancers, most notably in medulloblastoma, a primary brain tumor of childhood.^94^ Suggested mechanisms include KCTD11‐mediated suppression of the hedgehog signaling pathway by interacting with cullin‐3 via the BTB domain of KCTD11, while the KCTD11 C‐terminus recruits the Gli deacetylase HDAC1 for degradation.^12^ *KCTD11* is located on 17p13.1 near *TP53*. In an in vivo mouse screen to test the effect of haploinsufficiency of *TP53*‐linked genes, mouse *Kctd11* was identified as a tumor suppressor gene.^95^ However, neither of these findings has been confirmed by follow‐up investigations.

Until very recently, human KCTD11 was annotated in the NCBI and UniProt databases as a 232 amino acid protein with a truncated BTB domain (currently annotated at NCBI as KCTD11s, NP_001002914). However, coding sequencings for the missing N‐terminal segment of the BTB domain are present in‐frame immediately preceding the most 5‐prime ATG start of translation. In vitro studies suggest that KCTD11 is translated from an upstream non‐AUG (AUU) start codon (which may occur more commonly than appreciated), adding 39 amino acids to the N‐terminus of human KCTD11.^96^ NCBI recently revised the annotation of human KCTD11 as a 271 amino acid protein including a full BTB domain (KCTD11l, NP_001350571). A recent study showed that yeast Whi2 sharing a BTB domain homologous to that of human KCTD proteins is capable of inhibiting TORC1 under low amino acid conditions.^14^ Remarkably, human KCTD11 but not other KCTD proteins tested (KCTD7, KCTD8, KCTD11, KCTD12, and KCTD16) could suppress TORC1 activity when expressed in *whi2* ‐deficient yeast and in mammalian cell lines under low amino acid conditions.^14^ Furthermore, knockdown of *KCTD11* in HEK293 cells confirmed that KCTD11 is required to suppress mTORC1 during amino acid deprivation.^14^ The detailed molecular mechanism of how KCTD11 regulates mTORC1 activity is still unknown. One speculation is that there is crosstalk between mTORC1 and the hedgehog signaling pathways through KCTD11 in cancer.

### 4.2 KCTD15 in neural crest formation and obesity

Genome‐wide association studies (GWAS) have identified *KCTD15* variants in association with increased risk of obesity.^97^, ^98^ Although the detailed molecular mechanisms are not known, several lines of evidence suggest a potential role for *KCTD15* in obesity through inhibition of Wnt signaling. KCTD15 was reported to control/limit neural crest formation in zebrafish and frog embryos by attenuating the Wnt‐β‐catenin signaling pathway, as overexpression of *KCTD15* decreased neural crest formation while *KCTD15* knockdown caused neural crest size to increase.^99^ Follow‐up studies carried out by the same group showed that in zebrafish embryos and in human cells, KCTD15 directly inhibits the transcription factor AP‐2α, a target of Wnt signaling, consistent with a role for KCTD15 in neural crest development.^10^ The proposed mechanism is that KCTD15 binds to the proline‐rich activation domain of AP‐2α to prevent transcriptional activation by AP‐2α and that SUMO modification in the C‐terminus of zebrafish Kctd15 on Lys252 (human K278) inhibits the ability of KCTD15 to suppress transcription and inhibit neural crest formation.^10^, ^100^ Given that mesenchymal stem cells and some adipocytes are derived from the neural crest,^101^ and that AP‐2 regulates the expression of genes important for adipogenesis, such as *C/EBPα* and *IRS‐* 1,^102^, ^103^ it is conceivable that KCTD15 may regulate adipogenesis by regulating AP‐2 transcription activity in neural crest during development.

## 5 PERSPECTIVES

Several human *KCTD* family genes are expressed predominantly in the brain. Genetic alternations in *KCTD* family members have been associated with neurodevelopmental disorders, epilepsy, autism, schizophrenia, movement disorders, obesity, and several cancers, but little is understood about the biological functions of KCTD family proteins (Table 1). The original expectation that BTB domains of KCTD family members might directly partner with their nearest homologs, the T1/BTB domains of voltage‐gated potassium channels, currently lacks confirmation. However, new evidence supports roles for KCTDs in signaling pathways to indirectly modulate potassium channel activity. KCTDs appear to be involved in other processes, including nutrient sensing and autophagy, based on work in yeast showing that the yeast KCTD‐like protein Whi2 interacts with yeast phosphatases Psr1/Psr2 to regulate TORC1 activity.^14^ The plethora of effects of KCTD family proteins may reflect an adaptor function of KCTDs that recruits substrates for ubiquitination by cullin‐3 and subsequent degradation, though evidence also supports additional mechanisms. The highly variable C‐terminal regions of KCTD proteins could potentially reflect their roles in recruiting diverse substrates for cullin‐3‐mediated ubiquitination and degradation, though this is only one possibility. A binding site of the heterotrimeric G‐protein subunits Gβγ has been mapped to the C‐terminus of KCTD12 and KCTD16. Though it is not known whether this interaction serves only to desensitize the G‐protein coupled inwardly rectifying potassium channel GIRK or whether KCTD‐mediated protein turnover or other functions are involved. Defects in ubiquitination‐dependent protein function and/or GTPase modulated ion flux may underlie the neurodevelopmental and neuropsychiatric disorders associated with mutations affecting KCTD family proteins. In addition, more animal models are needed to understand pathophysiological roles of KCTDs before comprehending the detailed mechanisms of human disease.

Difficulties in diagnosing children with rare disease mutations in KCTD proteins have contributed to the misassignment of symptoms to other causes. The early‐onset age for autism and several other KCTD‐associated disorders coincide with the timing of childhood vaccinations. Consequently, patient narratives and social media indicate that these families spent years without an explanation for their child's illness. Patients with undiagnosed disease mutations seek explanations from circumstantial evidence. For example, until 2012 there was only a single publication implicating *KCTD7* mutations in disease.^38^ Similarly, a separate cohort of patients with encephalopathies attributed to vaccine reactions were instead due to de novo mutations in SCN1A.^104^, ^105^ Thus, from a policy perspective as well as from a therapeutic perspective, new knowledge about KCTD family protein functions is urgently needed.

## CONFLICT OF INTEREST

The authors have no conflicts of interest to declare.

## Supporting information

Table S1. BTB amino acid sequences used to generate figures 1 and 2.

Table S2. BTB alignment used for the analysis presented in figure 2.
