# Supplementary material for: KCTD: A new gene family involved in neurodevelopmental and neuropsychiatric disorders
Source: CNS Neurosci Ther. 2019 Jun 14;25(7):887–902. doi: 10.1111/cns.13156 (PMC6566181; doi:10.1111/cns.13156)
Supplement: Supplementary file 1 — Table S1. BTB amino acid sequences used to generate figures 1 and 2. [file CNS-25-887-s001.pdf]

>Q719H9_KCTD1HOMSA
PAQLTKSNAPVHIDVGGHMYTSSLATLTKYPESRIGRLFDGTEPIVLDSLKQHYFIDRDGQMFRYILNFL
RTSKLLIPDDFKDYTLLYEEAKYFQLQPMLLEMERWKQDRETG
>Q5M956_KCTD1MUSMU
PAQLTKSNAPVHIDVGGHMYTSSLATLTKYPESRIGRLDGTEPIVLDSLKQHYFIDRDGQMFRYILNFLR
TSKLLIPDDFKDYTLLYEEAKYFQLQPMLLEMERWKQDRETG
>Q5SNS9_DANRE
PAQLTKANAPVHIDVGGHMYTSSLATLTKYPESRIGRLFDGTEPIVLDSLKQHYFIDRDGHMFRYILNFL
RTSKLLIPDDFKDYSLLYEEARYFQLQPLQVELERWRSEQDSR
>Q14681_KCTD2HOMSA
RAGGGGAARWVRLNVGGTYFVTTRQTLGREPKSFLCRLCCQEDPELDSDKDETGAYLIDRDPTYFGPILN
YLRHGKLIITKELAEEGVLEEAEFYNIASLVRLVKERIRDNEN
>Q8CEZ0_KCTD2MUSMU
RTSGADGRRRGRPLGPAQRGRYLLRDTRQTLGREPKSFLCRLCCQEDPELDSDKDETGAYLIDRDPTYFG
PILNYLRHGKLIITKELGEEGVLEEAEFYNIASLVRLVKERIRDNEN
>Q0P490_DANRE
PGSRWVRLNVGGTYFVTTKQTLCRDPKSFLYRLCQEDPDLDSDKDETGAYLIDRDPTYFGPILNYLRHGK
LIINKNLAEEGVLEEAEFYNIASLVRLVKERIRDNEN
>Q9Y597_KCTD3HOMSA
MAGGHCGSFPAAAAGSGEIVQLNVGGTRFSTSRQTLMWIPDSFFSSLLSGRISTLRDETGAIFIDRDPAA
FAPILNFLRTKELDLRGVSINVLRHEAEFYGITPLVRRLLLCEELERSSCGSVL
>Q8BFX3_KCTD3MUSMU
MAGGHCGSFAASAASSGEIVQLNVGGTRFSTSRQTLMWIPDSFFSSLLSGRISTLRDETGAIFIDRDPAA
FAPILNFLRTKELDLRGVSINVLRHEAEFYGITPLVRRLLLCEELERSSCGSVL
>F1Q6W0_DANRE
MAGTGNSSTSGMGDIIQLNVGGTRFSTSRQTLTWIPDSFFSSLLSGRISTLRDETGAIFIDRDPTAFAPI
LNFLRTKELDLRGVNISILRHEAEFYGITPLVRRLLLCEELERSSCGSVL
>Q8WVF5_KCTD4HOMSA
GKNCKSTLMTLNVGGYLYITQKQTLTKYPDTFLEGIVNGKILCPFDADGHYFIDRDGLLFRHVLNFLRNG
ELLLPEGFRENQLLAQEAEFFQLKGLAEEVKSRWEKE
>Q9D7X1_KCTD4MUSMU
GKNCKSTLMTLNVGGYLYITQKQTLTKYPDTFLEGIVNGKILCPFDADGHYFIDRDGLLFRHVLNFLRNG
ELLLPEGFRENQLLAQEAEFFQLKGLAEEVKSRWEKE
>Q6GMI6_DANRE
SKSFKKPSSGTITINVGGYLYAAQRHTLAKHPGSLLEEMVTGKKPVLHVDSMGNTFIDRDGPIFRHILNF
LRLGELVLPEDFKETELLRREANFYRLSELAQALQDWEQQQ
>Q9TZA6_CAEEL
MCSSETIVKLNVGGSVFETWKSTLTKQDGFFKTLVETNIPVKKDTSDCYFIDRSPKYFETVLNYMRSGVT
VLPDSEKELQELKKEAEFYLLEQLVDLCEPINNQIRTYRSSHELL
>Q9NXV2_KCTD5HOMSA
AQRPGSVSKWVRLNVGGTYFLTTRQTLCRDPKSFLYRLCQADPDLDSDKDETGAYLIDRDPTYFGPVLNY
LRHGKLVINKDLAEEGVLEEAEFYNITSLIKLVKDKIRERDSKTSQVPVK
>Q8VC57_KCTD5MUSMU
AQRPGGVSKWVRLNVGGTYFLTTRQTLCRDPKSFLYRLCQADPDLDSDKDETGAYLIDRDPTYFGPVLNY
LRHGKLVINKDLAEEGVLEEAEFYNITSLIKLVKDKIRERDSRISQMPVK
>Q6NYY3_DANRE
APSPGERLQPSGSGSSKWVRLNVGGTYFLTTRQTLCRDPKSFLYRLCQADPDLDSDKDETGAYLIDRDPT
YFGPVLNYLRHGKLVLNRGLAEEGVLEEAEFYNITSLIKLVKDKIRERDCKTAQLPVK
>Q9W579_DROME
MSTVFINSRKSPNVLKKQGTDQWVKLNVGGTYFLTTKTTLSRDPNSFLSRLIQEDCDLISDRDETGAYLI

DRDPKYFAPVLNYLRHGKLVLDGVSEEGVLEEAEFYNVTQLIALLKECILHRDQRPQTDKKRVYRVLQCR
EQELTQM
>Q18776_CAEEL
MSSNTDDSTNQNGVAAVKTNGFHYGSSSSSWVRLNVGGKVFQTTRSTLMREPCSFLYRLCQDEMGLPTDR
DETGAYLIDRDPDFFSPILNYLRHGKLIMNPGLSEEGILAEADFYNLPSLSQLIMDRIQDRENSVKDATN
KFVYRVLQCHEEELASVV
>Q8NC69_KCTD6HOMSA
YMMTDPVTLNVGGHLYTTSLTTLTRYPDSMLGAMFGGDFPTARDPQGNYFIDRDGPLFRYVLNFLRTSEL
TLPLDFKEFDLLRKEADFYQIEPLIQCLNDPKPLYPM
>Q8BNL5_KCTD6MUSMU
MDNGDWGYMMSDPVTLNVGGHLYTTSLTTLTRYPDSMLGAMFGGDFPTARDPQGNYFIDRDGPLFRYVLN
FLRTSELTLPLDFKEFDLLRKEADFYQIEPLIQCLNDPRPLYPM
>Q6DG99_DANRE
MDNGDWGHRLTHPVTLNVGGHLYTTSISTLQRYPDSMLGAMFRGDFPTTRDAQGNYFIDRDGTLFRYILN
FLRTSELTLPVDFTELDLLRKEADFYQIEPLIQCLNDPKPLYPL
>Q9W2F9_DROME
AAASRYTAPVHIDVGGTIYTSSLETLTKYPESKLAKLFNGQIPIVLDSLKQHYFIDRDGGMFRHILNFMR
NSRLLIAEDFPDLELLLEEARYYEVEPMIKQLESMRKDRVRNGNYLVAPPTP
>Q96MP8_KCTD7HOMSA
LPLLPQEFPEVVPLNIGGAHFTTRLSTLRCYEDTMLAAMFSGRHYIPTDSEGRYFIDRDGTHFGDVLNFL
RSGDLPPRERVRAVYKEAQYYAIGPLLEQLENMQPLKGEKVRQ
>Q8BJK1_KCTD7MUSMU
LPLLPQEFPEVVPLNIGGAHFTTRLSTLRRYEDTMLAAMFSGRHYIPTDSEGRYFIDRDGTHFGDVLNFL
RSGDLPPREHVRAVHKEAQYYAIGPLLEQLENMQPLKGEKVRQ
>Q0VFV7_DANRE
TLDAPEEFPEVIPLNVGGTYFTTRLSTLRRYEDTMLAAMFSGRHHIPRDAEGRYFIDRDGTYFGDILNFL
REGELPQRDRVRAVHREAQYYAIGPLLENLEDTQPLTGEKVRQ
>Q7JZ62_DROME
MSESMSGDHKILLKGHSSQYLKLNVGGHLYYTTIGTLTKNNDTMLSAMFSGRMEVLTDSEGWILIDRCGN
HFGIILNYLRDGTVPLPETNKEIAELLAEAKYYCITELAISCERALYAHQEPKPICRIPLITSQKEEQLL
LSVSLKPAVILVVQRQNNKYSYTSTSDDNLLKNIEL
>Q18986_CAEEL
MEPSTIVKLDVGGKIFKTTIFTLCKHDSMLKTMFCTDVPVTKNEEGSVFIDRDSKHFRLILNFLRDGQIA
LPDSDREVREVLAEASYFLLDPLIELCGERLEQSLNPYYHLVSTVLEARKIIFATEKPIVVLRLPVYIAT
SGNQSYYFSETK
>Q6ZWB6_KCTD8HOMSA
GPCAPSPFPEVVELNVGGQVYVTKHSTLLSVPDSTLASMFSPSSPRGGARRRGELPRDSRARFFIDRDGF
LFRYVLDYLRDKQLALPEHFPEKERLLREAEYFQLTDLVKLLSPKVTKQNS
>Q50H33_KCTD8MUSMU
GPCAPSPFPEVVELNVGGQVYVTKHSTLLSVPDSTLASMFSPSSPRGGARRRGDLPRDSRARFFIDRDGF
LFRYVLDYLRDKQLALPEHFPEKERLLREAEFFQLTDLVKLLSPKVTKQNS
>Q3L1G3_DANRE
MAMKETILPISEVSSPYPEVVELNVGGQVYVTKRSTLVSVPDTTLHTMFTRCTPHELPRDSRGRFFIDRD
GFLFRYVLDFLRDRQLVLPEHFPERERLQREAEHFQLGELLRLLGPRVAKQGS
>A0PD34_CAEEL
MCAVPFLGASIYIYILPNRAYYMTSVEDVITLNVGGTMYTTTRSTLSKETDTLLANIASGSLSEDEQANV
VTLPDGTLFVDRDGPLFAYVLHFLRTDKLSLPEQFREVARLKDEADFYRLERFSTLLSNASSISPRPRTA
NGYNTITSGAETGGYITLGYRGT
>Q7L273_KCTD9HOMSA
PEGLLGFHTDWLTLNVGGRYFTTTRSTLVNKEPDSMLAHMFKDKGVWGNKQDHRGAFLIDRSPEYFEPIL

NYLRHGQLIVNDGINLLGVLEEARFFGIDSLIEHLEVAIKNSQPPEDHSPIS
>Q80UN1_KCTD9MUSMU
PGGLLGSHTDWLTLNVGGRYFTTTRSTLVNKEPDSMLAHMFKDKGVWGNKQDHRGAFLIDRSPEYFEPIL
NYLRHGQLIVNDGINLLGVLEEARFFGIDSLIEHLEVAIKNSQPPEDHSPIS
>Q6DGD4_DANRE
KGGLIDDIALIRDDDVLYISEGDSFVDPPNNPESVHEFNSWAHTDWITLNVGGRRFTTTRSTLVKETESM
LAHMFRDKDVWGNKQDEQGAYLIDRSPDYFEPILNYLRHGQLIINDGINLLGVLEEARFFGIERLAEQLE
GVIKNSQPPDDHSPIS
>F1Q5M1_DANRE
KGGLIDDIALIRDDDVLYVSEGDAFIDPHSEGKMSDDISGSHTDWLTLNIGGRLFTTTRSTLVSKEPDSM
LAHMFREKDVWGNKQDERGAFLIDRSPEYFEPILNYLRHGQIIINDGINLLGVLEEARFFGIEQLAEQLE
VAIKNSHPPEDHSPLS
>Q8T0F7_DROME
MTGEEREDKAAENCQALWKFFKLPNQMNSDLKESGSGARKDDAAPAAGDFVAASGFAPNRWVKLNVGGQI
YATTIDTLVGREPDSMLARMFLQNGSMKPSERDEQGAYLIDRSPRYFEPIINYLRHGQFVCDSNISVLGV
LEEARFFGIFSLVTHLEERLGQQETPLGDRPLT
>Q9H3F6_KCTD10HOMSA
MEEMSGESVVSSAVPAAATRTTSFKGTSPSSKYVKLNVGGALYYTTMQTLTKQDTMLKAMFSGRMEVLTD
SEGWILIDRCGKHFGTILNYLRDGAVPLPESRREIEELLAEAKYYLVQGLVEECQAALQNKDTYEPFCKV
PVITSSKEEQKL
>Q922M3_KCTD10MUSMU
MEEMSGDSVVSSAVPAAATRTTSFKGASPSSKYVKLNVGGALYYTTMQTLTKQDTMLKAMFSGRMEVLTD
SEGWILIDRCGKHFGTILNYLRDGGVPLPESRREIEELLAEAKYYLVQGLLEECQAALQNKDTYEPFCKV
PVITSSKEEQRL
>Q6P7X5_DANRE
MEEMSGESVVSSAVPAATTRTTSFKGSSPSSKYVKLNVGGALYYTTMQTLTKQDTMLKAMFSGRMEVLTD
SEGWILIDRCGKHFGTILNYLRDGVVPLPESRRETEELLAEAKYYLVQGLVDECQAALQNKDAYEPFCKV
PLVTSSKEEQRL
>Q693B1_KCTD11HOMSA
ISPPPVPSSPPSFGGPVTLNVGGTLYSTTLETLTRFPDSMLGAMFRAGTPMPPNLNSQGGGHYFIDRDGK
AFRHILNFLRLGRLDLPRGYGETALLRAEADFYQIRPLLDALRELEASQGTPAPTAALLHADVDVSPRLV
HFSARRGPHHYELSSVQVDTFRAN
>Q8K485_KCTD11MUSMU
TLPPNSFQTPKYLLFPSPCLSLILPKISPPPVSSLPPSFGGPVTLNVGGTLYSTTLETLTRFPDSMLGAM
FRADTLMPANLNPQGDGHYFIDRDGKAFRHILNFLRLGRLDLPRGYGETALLKAEADFYQIRPLLDALRE
LEASRGTPASTAALLHADVDVSPRQVHFSARRGPHHYELSSVQVDTFRAN
>Q96CX2_KCTD12HOMSA
MALADSTRGLPNGGGGGGGSGSSSSSAEPPLFPDIVELNVGGQVYVTRRCTVVSVPDSLLWRMFTQQQPQ
ELARDSKGRFFLDRDGFLFRYILDYLRDLQLVLPDYFPERSRLQREAEYFELPELVRRLGAPQQPGPGPP
PSRRGVHKEG
>Q6WVG3_KCTD12MUSMU
MALADSARGLPNGGGGGGGSGSSSSSAEPPLFPDIVELNVGGQVYVTRRCTVVSVPDSLLWRMFTQQQPQ
ELARDSKGRFFLDRDGFFFRYILDYLRDLQLVLPDYFPERSRLQREAEYFELPELVRRLGAPQQPGPGPP
PPHSRRGVHKEG
>Q804Q4_DANRE
MALADPECGISNGADSASPFSVIIELNVGGQVYVTRHTTLIAVPDSLLWNMFSKKTPAELARDSKGRFFL
DRDGFLFRYILDYLRDLNLVLPDYFPEKSRLQREAEFFQLRDLSKLLSPKMSKDNSITDEICQSDSEEPS
ASATPVVGPETSRTLSVASSAHSPSLESRKSGYITVGYRGSYTMG
>Q561T3_DANRE
MDKTRADASSPRFSEIIELNVGGQVYVTRHSTLLSVPNSLLWTMFSQKKPAELTTDSKGRFFLDRDGFLF

RYILDYLRDQTLVLPDYFKEKASLLKEAEYFQLQDLAKRLKPAVSKENSISEEVCQSDPEEAALAGTSMT
CTGPRSPSLDARKTGFITIGYRGSYTIG
>Q9VDH3_DROME
MPEIIELNVGGVSYTTTLATLLQDKSTLLAELFGEGRDSLAKDSKGRYFLDRDGVLFRYILDFLRDKALH
LPEGFRERQRLLREAEHFKLTAMLECIRSERDARPPGCITIGYRGSFQFGKDGLADVKFRKLSRILVCGR
>Q8WZ19_KCTD13HOMSA
MSAEASGPAAAAAPSLEAPKPSGLEPGPAAYGLKPLTPNSKYVKLNVGGSLHYTTLRTLTGQDTMLKAMF
SGRVEVLTDAGGWVLIDRSGRHFGTILNYLRDGSVPLPESTRELGELLGEARYYLVQGLIEDCQLALQQK
RETLSPLCLIPMVTSPREEQQ
>Q8BGV7_KCTD13MUSMU
MSAEASGPAPAAAECLESPSPSSVEPGSPSYSLKPLTPNSKYVKLNVGGSLHYTTLRTLTGQDTMLKAMF
SGRVEVLTDAGGWVLIDRSGRHFGTILNYLRDGSVPLPESARELGELLGEARYYLVQGLIEDCQLALQQK
REKLSPLCLIPTVTSPREEQQ
>A9ULR9_DANRE
MSAEASGSSGGHAVTVSGSSPSSSSHVGEEKPGRSLVSSKYVKLNVGGTLHYTTVQTLSKEDSLLRSICD
GSTEVSIDSEGWVVLDRCGRHFSLVLNFLRDGTVPLPDSTRELEEVLKEAQYYRLQGLVQHCLSTLQKRR
DVCRGCHIPMITSAKEEQR
>Q9BQ13_KCTD14HOMSA
MWQGCAVERPVGRMTSQTPLPQSPRPRRPTMSTVVELNVGGEFHTTTLGTLRKFPGSKLAEMFSSLAKAS
TDAEGRFFIDRPSTYFRPILDYLRTGQVPTQHIPEVYREAQFYEIKPLVKLLEDMPQIFGEQVSRKQFLL
QVPGYSENLE
>Q5EER8_KCTD14MUSMU
MSSWAKVIYGASTELDAKVVELNVGGQFYTTTMGTLMKHPGSKFSEILSRSARHYKDAQGRFFIDRPGTY
FGLLLDYLRTGQVPTEYVPEVYQEAKFYQIHLLVKILEDMPQIFGEQVARTQFLMGVPNYRENLE
>A0A2R8RRJ7_DANRE
MSLPDYKTIGKQSSAPVQSKSQVVHLNIGGHVFSTTLGTIRKFPNSTLAELINGSSKRMDSEGRYFIDRD
GTLFTHILEYLRTEKLPCEHLQEVHKEAIYYDIKPLVKAIEETPQFFGETVGRQQFLARVPNYRENLE
>Q96SI1_KCTD15HOMSA
QGIPLPAQLTKSNAPVHIDVGGHMYTSSLATLTKYPDSRISRLFNGTEPIVLDSLKQHYFIDRDGEIFRY
VLSFLRTSKLLLPDDFKDFSLLYEEARYYQLQPMVRELERWQQEQEQRRRSRACDCLVVRV
>Q8K0E1_KCTD15MUSMU
QGIPLPAQLTKANAPVHIDVGGHMYTSSLATLTKYPDSRISRLFNGTEPIVLDSLKQHYFIDRDGEIFRY
ILSFLRTSKLLLPDDFKDFNLLYEEARYYQLQPMVRELERWQQDQEQRRRSRACDCLVVRVTPDLGER
>Q6DC02_DANRE
QGIPLPAQLTKANAPVHIDVGGHMYTSSLATLTKYPDSRISRLFNGTEPIVLDSLKQHYFIDRDGEIFRY
ILSFLRTCKLLLPDDFKDFNLLYEEARYYQLSPMIKELERWKQEREQRRLANPCDCLVVRVTPDLGER
>Q68DU8_KCTD16HOMSA
PREQGSAVPNSFPEVVELNVGGQVYFTRHSTLISIPHSLLWKMFSPKRDTANDLAKDSKGRFFIDRDGFL
FRYILDYLRDRQVVLPDHFPEKGRLKREAEYFQLPDLVKLLTPDEIKQSPDEFCH
>Q5DTY9_KCTD16MUSMU
PRDQGAAVPNSFPEVIELNVGGQVYFTRHSTLISIPHSLLWKMFSPKRDTANDLAKDSKGRFFIDRDGFL
FRYILDYLRDRQVVLPDHFPERGRLKREAEYFQLPDLVKLLAPEDVKQSPDEFCH
>Q3L1G6_DANRE
PKDSGCAQSCSSDVVELNVGGQVYYTRHATLTSVPNSLLGKLFSSKKDISNDLTQDIKGRYFIDRDGFLF
RYVLDYLRDKTVVLPDYFPEKGRLKREAEFFQLPELVKILTPDDYSHS
>Q3L1G4_DANRE
MALSGNSKPKDSSFPDVIELNVGGQVYYTRYTTLINTPGSLLGKIFSPKNNASNDLARDPKGRYFIDRDG
FLFRYVLDYLRDKQVVLPDHFPEKGRLRKEAEYFQLPDLVKLLTPDDLKPSSDEYIH
>Q8N5Z5_KCTD17HOMSA
PAGAGGRAAGGWGKWVRLNVGGTVFLTTRQTLCREQKSFLSRLCQGEELQSDRDETGAYLIDRDPTYFGP

ILNFLRHGKLVLDKDMAEEGVLEEAEFYNIGPLIRIIKDRMEEKDYTVTQVPPKHVYRV
>A6H697_KCTD17MUSMU
PVGAGGRPGGGWGKWVRLNVGGTVFLTTRQTLCREQKSFLSRLCQGEELQSDRDETGAYLIDRDPTYFGP
ILNFLRHGKLVLDKDMAEEGVLEEAEFYNIGPLIRIIKDRMEEKDYTVAQVPPKHVYRV
>U3JA92_DANRE
DNEGSEGATTTTSTDVESTGAENIIGNGSAVNTTGGNNGKWVRLNVGGTVFLTTRQTLLKEQTSFLYRLC
QQQDLHSDTDETGAYVIDRDPTYFGPILNYLRHGKLVYNKELAEEGVLEEAEFYNITPLIKLIKERILER
DSKATQQVPPKHVYRV
>Q6PI47_KCTD18HOMSA
MEGHKAEEEVLDVLRLNVGGCIYTARRESLCRFKDSMLASMFSGRFPLKTDESGACVIDRDGRLFKYLLD
YLHGEVQIPTDEQTRIALQEEADYFGIPYPYSLSDHLANEMET
>Q6DI85_KCTD18MUSMU
MAGHEAEDVLDILRLNVGGCIYTARRESLCRFKDSMLASMFSGRFPLKTDESGACIINRDGHLFKYILDY
LHGEVQTPSDEQTRAALQEEADYFGIPYPYSLSDHLANEMETYSLRL
>Q17RG1_KCTD19HOMSA
WTAEITVYSPQQIIKVYVGSHWYATTLQTLLKYPELLSNPQRVYWITYGQTLLIHGDGQMFRHILNFLRL
GKLFLPSEFKEWPLFCQEVEEYHIPSLSEALAQCEAYKSWTQEKESENEE
>Q562E2_KCTD19MUSMU
WTAEVTIYSPQQIIKLYVGSHWYATTLQTLMKYPELLSNTQRVYWIAYGQTLLIHGDGQMFRHILNFLRL
GKLFLPSEFKEWPLFCQEVEEYHIPALSEALAQCEAYKSWTQEKESENEEAFPIRKLHVVTEGTGP
>Q7Z5Y7_KCTD20HOMSA
SVGFGSNSHSQAPEKVTLLVDGTRFVVNPQIFTAHPDTMLGRMFGPGREYNFTRPNEKGEYEIAEGISAT
VFRTVLDYYKTGIINCPDGISIPDLRDTCDYLCINFDFNTIRCQDLSAL
>Q8CDD8_KCTD20MUSMU
SHEPFIVPERFGNSGLGFGGGAHSQAPEKVTLLVDGTRFVVNPQIFTAHPDTMLGRMFGPGREYNFTRPN
EKGEYVIAEGISATVFRTVLDYYKTGIINCPDGISIPDLRDTCDYLCINFDFNTIRCQDLSAL
>A0A2R8QE47_DANRE
PRPLSHDRVTLVVDGTHFVVDPAVFTAYPDTVLGRMFGRARQHSFTRPNTKGEYEIAEGIGASIFRIILD
FYRVGILHCPEGVSLAELREACDYLCINFDYNTVRCRDLSALLHEL
>X2J8G3_DROME
MSGGGAVPKHDIKSPCGASPSCQATGSGMGRLPLPPERITMLVDGVRFTVEQSLLTAHPTTMLGTMFGSG
FQFAHTNERGEYDVADGISHLVFRAILEYYKSGVIRCPPTVSVPELKEACDYLLIPFDATTVRCQNLSL
>Q21748_CAEEL
EGDKVCLLVDQTRFLVSQRLLTSKPDTMLGRMFSMRASCGDLGADLVSPNERDEFEVADGMTSSCFRAIL
DYYQSGTMRCPSSVSVSELREACDYLLVPFNAQTVKCQNLHALLHEL
>Q4G0X4_KCTD21HOMSA
MSDPITLNVGGKLYTTSLATLTSFPDSMLGAMFSGKMPTKRDSQGNCFIDRDGKVFRYILNFLRTSHLDL
PEDFQEMGLLRREADFYQVQPLIEALQEKEVELSKAEKNAMLNI
>Q3URF8_KCTD21MUSMU
MSDPITLNVGGKLYTTSLATLTSFPDSMLGAMFSGKMPTKRDSQGNCFIDRDGKVFRYILNFLRTSHLDL
PEDFQEMGLLRREADFYQVQPLIEALQEKEVELSKAEKNAMLNI
>Q8TBC3_SHKB1HOMSA
MAAAATAAEGVPSRGPPGEVIHLNVGGKRFSTSRQTLTWIPDSFFSSLLSGRISTLKDETGAIFIDRDPT
VFAPILNFLRTKELDPRGVHGSSLLHEAQFYGLTPLVRRLQLREELDRSSCGNVLFNGYLPPPVFPVKRR
NRHSLVGPQQ
>Q6P7W2_SHKB1MUSMU
MAVATTAVEGVPSRGAPGEVIHLNVGGKRFSTSRQTLTWIPDSFFSSLLSGRISTLKDETGAIFIDRDPT
VFAPILNFLRTKELDPRGVHGSSLLHEAQFYGLTPLVRRLQVREELDRSSCGNVLFNGYLPPPVFPVKRR
NRHSLVGPQQ
>E7FFI2_DANRE

```
MARIGDIIHLNVGGKRFSTSRQTLTWVPDSFFSSLLSGRISTLKDETGAIFIDRDPSLFAPILNFLRTKE
LHPRSIDVHLLIHEAEFYGITPLVRKLQLCDELDRSSCGNVLFNGYLPPPVYPVKRRNRHSVAGPQF
>Q9VH62_DROME
MSHFTHASSSDLVNLNVGGQRFSTSRQTLTWIPDTFFTALLSGRISSLRDEHNAIFIDRDPTLFSIILNY
LRTKDIDIKNCEIRALRHEAEYYGITPLTKRLALCEDLNHSSCGDLLFYGFLAAPPMPSNEAVAATSVDE
SLPSTSASAIGSRPGSMVRVPEPSRSSHSRNSSWD
>O17001_CAEEL
MAHQPCSSSSFSNDSGSDYIVNLNVGGRIFATSCNTLTWIPDSFFTSLLSGRMNSVKDPSGAIFIDRDPD
LFRVILNYLRTKQVDLCGIKVDTLKHEALFFGLTPLIRRLTLCEELSSTSCGSVYFCGMIPPPDMPLENL
PNEKSRSPMYSNSSETSTNNSKNKSNGSPRHRGHSKKQSTDMSRYIKNEL
>Q13829_TNFAIP1HOMSA BACD2HOMSA
MSGDTCLCPASGAKPKLSGFKGGGLGNKYVQLNVGGSLYYTTVRALTRHDTMLKAMFSGRMEVLTDKEGW
ILIDRCGKHFGTILNYLRDDTITLPQNRQEIKELMAEAKYYLIQGLVNMCQSALQDKKDSYQPVCNIPII
TSLKEEERLI
>O70479_TNFAIP1MUSMU BACD2MUSMU
MSGDTCLCPASGAKPKISGFKGGGLGNKYVQLNVGGSLYYTTVRALTRHDTMLKAMFSGRMEVLTDKEGW
ILIDRCGKHFGTILNYLRDDTITLPQSRQEIQELMAEAKYYLIQGLVSTCQTALQDKKDSYQPVCNIPII
TSLREEDRLI
>F1R5K5_DANRE
MSGESCLHQLQPHTGPILPVSSVGCPKTNTCTYRGVTGNKYVQLNVGGNLYYSTLQVLTRQDTLLRSMFS
GKMEVLTDKEGWILIDRCGKHFGSILSYLRDGFVNLPKSRQSIMELLAEAKYYQIQGLIDLCQKELQDNK
EKALCVIPVITSPKEEERLI
>P12611_WHI2SACCE
QNSQYEGNEEDYGDSLIHLNIQENHYFITRDQLMSLPESLLLCLFPSGVFLDRCGQVITNLTRDDEVYIV
NFPPDCFEYIMEIYTKAHDDLYNHPVEKFFDRPSSSFVSNAKGFFGLSSNNSISSNNEQDILHQKPAIIV
LREDLDYYCVPQEEFQFDSTNEENNEDLLRHFMAQVKMAA
>Q9P7D3_WHI2SACPO
MSVITQAQEIPTVAANPDFNYEGDPIIIQLCDRDTVFELSRDQLLGLPESILMCLFPRGLLLDYEIQECQ
LTQRPLIFQTADFDPSLLQYILNYFQMAENRTANDEIALSPPPPSFPGKCGIILLKEDIEFFILPPISPT
TNIAIEVSPNDLLKLKQRVAQRLLQQKK
>A0A1D8PQP1_WHI2CANAL
MSTSTSSQQQPQDIITQVSPHQQDSFNTTTTTHTTGTDDQSDVGGGGDYNSIIHLNIRGKEFTITRDDLM
SLPESILLCLFPNGVFLDVNGNVINNLTEDDIVYVNFDPQTFQYIINTFYQAQQDLIQMSTNTTNLTPVT
SHNNGRHNNNNNNSSRHNRQENILETKPAIIVLREDLDFYVIPPFERLNSEQMKQLKLGVSIQLLKNKL
>Q2TUM3_KCNRG_MUSMU
GQDLVTLNVGGRIFTTRPSTLKQFPASRLAGMLDGRDQEFKTVDGQIFVDRDGALFSFILDFLRNHELLL
PSDFADHHRLQREALFYELDSLVDLLSQFLLQSRSAVME
>Q8N5I3_KCNRG_HOMSA
SQELVTLNVGGKIFTTRFSTIKQFPASRLARMLDGRDQEFKMVGGQIFVDRDGDLFSFILDFLRTHQLLL
PTEFSDYLRLQREALFYELRSLVDLLNPYLLQPRPALVE
>Q9BSF8_BTBDAHOMSA
TSERVTLIVDNTRFVVDPSIFTAQPNTMLGRMFGSGREHNFTRPNEKGEYEVAEGIGSTVFRAILDYYKT
GIIRCPDGISIPELREACDYLCISFEYSTIKCRDLSALMHEL
>Q80X66_BTBDAMUSMU
TSERVTLIVDNTRFVVDPSIFTAQPNTMLGRMFGSGREHNFTRPNEKGEYEVAEGIGSTVFRAILDYYKT
GIIRCPDGISIPELREACDYLCISFEYSTIKCRDLSALMHEL
>A4IG48_DANRE
TAERVTLIVDNTRFVVDPSIFTAQPNTMLGRMFGSGREHNFTRPNEKGEYEVAEGISSTVFRAILDYYKS
GIIRCPDGISIPELREACDYLCIAFDYSTIKCRDLSALMHEL
>X1WDG9_DANRE
```

TSEKVTLIVDNTRFVVDPSIFTAQPNTMLGRMFGSGREHNFTRPNEKGEYEVAEGISSTVFRAILDYYKS
GIIRCPDGISIPELREACDYLCISFDYSTIKCRDLSALMHEL
