# Supplementary material for: KCTD: A new gene family involved in neurodevelopmental and neuropsychiatric disorders
Source: CNS Neurosci Ther. 2019 Jun 14;25(7):887–902. doi: 10.1111/cns.13156 (PMC6566181; doi:10.1111/cns.13156)
Supplement: Supplementary file 2 — Table S2. BTB alignment used for the analysis presented in figure 2. [file CNS-25-887-s002.pdf]

CLUSTAL W (1.7) multiple sequence alignment

```
KCTD1_HOMSA         SNAPVHIDVGGHMYTSSLATLT-KYPESRIGRLFDGTEPIVL--------
DSLKQHYFID
KCTD1_MUSMU         SNAPVHIDVGGHMYTSSLATLT-KYPESRIGRL-DGTEPIVL--------
DSLKQHYFID
Q5SNS9_DANRE        ANAPVHIDVGGHMYTSSLATLT-KYPESRIGRLFDGTEPIVL--------
DSLKQHYFID
KCTD15_HOMSA        SNAPVHIDVGGHMYTSSLATLT-KYPDSRISRLFNGTEPIVL--------
DSLKQHYFID
KCTD15_MUSMU        ANAPVHIDVGGHMYTSSLATLT-KYPDSRISRLFNGTEPIVL--------
DSLKQHYFID
Q6DC02_DANRE        ANAPVHIDVGGHMYTSSLATLT-KYPDSRISRLFNGTEPIVL--------
DSLKQHYFID
Q9W2F9_DROME        YTAPVHIDVGGTIYTSSLETLT-KYPESKLAKLFNGQIPIVL--------
DSLKQHYFID
KCTD6_HOMSA         MTDPVTLNVGGHLYTTSLTTLT-RYPDSMLGAMFGGDFPTA---------
RDPQGNYFID
KCTD6_MUSMU         MSDPVTLNVGGHLYTTSLTTLT-RYPDSMLGAMFGGDFPTA---------
RDPQGNYFID
Q6DG99_DANRE        LTHPVTLNVGGHLYTTSISTLQ-RYPDSMLGAMFRGDFPTT---------
RDAQGNYFID
KCTD21_HOMSA        MSDPITLNVGGKLYTTSLATLT-SFPDSMLGAMFSGKMPTK---------
RDSQGNCFID
KCTD21_MUSMU        MSDPITLNVGGKLYTTSLATLT-SFPDSMLGAMFSGKMPTK---------
RDSQGNCFID
KCTD11_HOMSA        FGGPVTLNVGGTLYSTTLETLT-RFPDSMLGAMFRAGTPMP-----
PNLNSQGGGHYFID
KCTD11_MUSMU        FGGPVTLNVGGTLYSTTLETLT-RFPDSMLGAMFRADTLMP-----
ANLNPQGDGHYFID
KCTD4_HOMSA         KSTLMTLNVGGYLYITQKQTLT-KYPDTFLEGIVNGKILCP---------
FDADGHYFID
KCTD4_MUSMU         KSTLMTLNVGGYLYITQKQTLT-KYPDTFLEGIVNGKILCP---------
FDADGHYFID
Q6GMI6_DANRE        SSGTITINVGGYLYAAQRHTLA-KHPGSLLEEMVTGKKPV--------
LHVDSMGNTFID
KCTD8_HOMSA         FPEVVELNVGGQVYVTKHSTLL-
SVPDSTLASMFSPSSPRGGARRRGELPRDSRARFFID
KCTD8_MUSMU         FPEVVELNVGGQVYVTKHSTLL-
SVPDSTLASMFSPSSPRGGARRRGDLPRDSRARFFID
Q3L1G3_DANRE        YPEVVELNVGGQVYVTKRSTLV-SVPDTTLHTMFTRCTP-------
HELPRDSRGRFFID
KCTD16_HOMSA        FPEVVELNVGGQVYFTRHSTLI-SIPHSLLWKMFSPKRDT-----
ANDLAKDSKGRFFID
KCTD16_MUSMU        FPEVIELNVGGQVYFTRHSTLI-SIPHSLLWKMFSPKRDT-----
ANDLAKDSKGRFFID
Q3L1G4_DANRE        FPDVIELNVGGQVYYTRYTTLI-NTPGSLLGKIFSPKNNA-----
SNDLARDPKGRYFID
Q3L1G6_DANRE        SSDVVELNVGGQVYYTRHATLT-SVPNSLLGKLFSSKKDI-----
```

SNDLTQDIKGRYFID
Q804Q4_DANRE FSVIIELNVGGQVYVTRHTTLI-AVPDSLLWNMFSKKTP-------
AELARDSKGRFFLD
Q561T3_DANRE FSEIIELNVGGQVYVTRHSTLL-SVPNSLLWTMFSQKKP-------
AELTTDSKGRFFLD
KCNRG_MUSMU GQDLVTLNVGGRIFTTRPSTLK-QFPASRLAGMLDGRDQ---------
EFKTVDGQIFVD
KCNRG_HOMSA SQELVTLNVGGKIFTTRFSTIK-QFPASRLARMLDGRDQ---------
EFKMVGGQIFVD
KCTD12_HOMSA FPDIVELNVGGQVYVTRRCTVV-SVPDSLLWRMFTQQQP-------
QELARDSKGRFFLD
KCTD12_MUSMU FPDIVELNVGGQVYVTRRCTVV-SVPDSLLWRMFTQQQP-------
QELARDSKGRFFLD
Q9VDH3_DROME MPEIIELNVGGVSYTTTLATLL-QDKSTLLAELFGEGR--------
DSLAKDSKGRYFLD
A0PD34_CAEEL VEDVITLNVGGTMYTTTRSTLS-KETDTLLANIASGSLSEDE---
QANVVTLPDGTLFVD
KCTD7_HOMSA FPEVVPLNIGGAHFTTRLSTLR-CYEDTMLAAMFSGRHYIP---------
TDSEGRYFID
KCTD7_MUSMU FPEVVPLNIGGAHFTTRLSTLR-RYEDTMLAAMFSGRHYIP---------
TDSEGRYFID
Q0VFV7_DANRE FPEVIPLNVGGTYFTTRLSTLR-RYEDTMLAAMFSGRHHIP---------
RDAEGRYFID
KCTD14_HOMSA MSTVVELNVGGEFHTTTLGTLR-KFPGSKLAEMFSSLAKAS---------
TDAEGRFFID
KCTD14_MUSMU DAKVVELNVGGQFYTTTMGTLM-KHPGSKFSEILSRSARHY---------
KDAQGRFFID
A0A2R8RRJ7_DANRE KSQVVHLNIGGHVFSTTLGTIR-KFPNSTLAELINGSSKR----------
MDSEGRYFID
KCTD2_HOMSA AARWVRLNVGGTYFVTTRQTLG-REPKSFLCRLCCQEDPE------
LDSDKDETGAYLID
Q0P490_DANRE GSRWVRLNVGGTYFVTTKQTLC-RDPKSFLYRLC-QEDPD------
LDSDKDETGAYLID
KCTD5_HOMSA VSKWVRLNVGGTYFLTTRQTLC-RDPKSFLYRLC-QADPD------
LDSDKDETGAYLID
KCTD5_MUSMU VSKWVRLNVGGTYFLTTRQTLC-RDPKSFLYRLC-QADPD------
LDSDKDETGAYLID
Q6NYY3_DANRE SSKWVRLNVGGTYFLTTRQTLC-RDPKSFLYRLC-QADPD------
LDSDKDETGAYLID
KCTD2_MUSMU GRPLGPAQRGRYLLRDTRQTLG-REPKSFLCRLCCQEDPE------
LDSDKDETGAYLID
KCTD17_HOMSA WGKWVRLNVGGTVFLTTRQTLC-REQKSFLSRLCQGEE--------
LQSDRDETGAYLID
KCTD17_MUSMU WGKWVRLNVGGTVFLTTRQTLC-REQKSFLSRLCQGEE--------
LQSDRDETGAYLID
U3JA92_DANRE NGKWVRLNVGGTVFLTTRQTLL-KEQTSFLYRLCQQQD--------
LHSDTDETGAYVID
Q18776_CAEEL SSSWVRLNVGGKVFQTTRSTLM-REPCSFLYRLCQ-DEMG------
LPTDRDETGAYLID
Q9W579_DROME TDQWVKLNVGGTYFLTTKTTLS-RDPNSFLSRLIQ-EDCD------

```
LISDRDETGAYLID
KCTD9_HOMSA        HTDWLTLNVGGRYFTTTRSTLVNKEPDSMLAHMFKDKGVW-------
GNKQDHRGAFLID
KCTD9_MUSMU        HTDWLTLNVGGRYFTTTRSTLVNKEPDSMLAHMFKDKGVW-------
GNKQDHRGAFLID
F1Q5M1_DANRE       HTDWLTLNIGGRLFTTTRSTLVSKEPDSMLAHMFREKDVW-------
GNKQDERGAFLID
Q6DGD4_DANRE       HTDWITLNVGGRRFTTTRSTLV-KETESMLAHMFRDKDVW-------
GNKQDEQGAYLID
Q8T0F7_DROME       PNRWVKLNVGGQIYATTIDTLVGREPDSMLARMFLQNGSMK------
PSERDEQGAYLID
KCTD3_HOMSA        SGEIVQLNVGGTRFSTSRQTLM-WIPDSFFSSLLSGRISTL---------
RDETGAIFID
KCTD3_MUSMU        SGEIVQLNVGGTRFSTSRQTLM-WIPDSFFSSLLSGRISTL---------
RDETGAIFID
F1Q6W0_DANRE       MGDIIQLNVGGTRFSTSRQTLT-WIPDSFFSSLLSGRISTL---------
RDETGAIFID
SHKB1_HOMSA        PGEVIHLNVGGKRFSTSRQTLT-WIPDSFFSSLLSGRISTL---------
KDETGAIFID
SHKB1_MUSMU        PGEVIHLNVGGKRFSTSRQTLT-WIPDSFFSSLLSGRISTL---------
KDETGAIFID
E7FFI2_DANRE       IGDIIHLNVGGKRFSTSRQTLT-WVPDSFFSSLLSGRISTL---------
KDETGAIFID
Q9VH62_DROME       SSDLVNLNVGGQRFSTSRQTLT-WIPDTFFTALLSGRISSL---------
RDEHNAIFID
O17001_CAEEL       SDYIVNLNVGGRIFATSCNTLT-WIPDSFFTSLLSGRMNSV---------
KDPSGAIFID
Q9TZA6_CAEEL       SETIVKLNVGGSVFETWKSTLT-K-QDGFFKTLVETNIPVK---------
KDTSDCYFID
Q18986_CAEEL       PSTIVKLDVGGKIFKTTIFTLC-K-HDSMLKTMFCTDVPVT---------
KNEEGSVFID
Q7JZ62_DROME       SSQYLKLNVGGHLYYTTIGTLT-KNNDTMLSAMFSGRMEVL---------
TDSEGWILID
KCTD10_HOMSA       SSKYVKLNVGGALYYTTMQTLT-K-QDTMLKAMFSGRMEVL---------
TDSEGWILID
KCTD10_MUSMU       SSKYVKLNVGGALYYTTMQTLT-K-QDTMLKAMFSGRMEVL---------
TDSEGWILID
Q6P7X5_DANRE       SSKYVKLNVGGALYYTTMQTLT-K-QDTMLKAMFSGRMEVL---------
TDSEGWILID
TNFAIP1_HOMSA      GNKYVQLNVGGSLYYTTVRALT-R-HDTMLKAMFSGRMEVL---------
TDKEGWILID
TNFAIP1_MUSMU      GNKYVQLNVGGSLYYTTVRALT-R-HDTMLKAMFSGRMEVL---------
TDKEGWILID
F1R5K5_DANRE       GNKYVQLNVGGNLYYSTLQVLT-R-QDTLLRSMFSGKMEVL---------
TDKEGWILID
KCTD13_HOMSA       NSKYVKLNVGGSLHYTTLRTLT-G-QDTMLKAMFSGRVEVL---------
TDAGGWVLID
KCTD13_MUSMU       NSKYVKLNVGGSLHYTTLRTLT-G-QDTMLKAMFSGRVEVL---------
TDAGGWVLID
A9ULR9_DANRE       SSKYVKLNVGGTLHYTTVQTLS-K-EDSLLRSICDGSTEVS---------
```

IDSEGWVVLD
KCTD18_HOMSA   VLDVLRLNVGGCIYTARRESLC-RFKDSMLASMFSGRFPLK---------
TDESGACVID
KCTD18_MUSMU   VLDILRLNVGGCIYTARRESLC-RFKDSMLASMFSGRFPLK---------
TDESGACIIN
KCTD19_HOMSA   PQQIIKVYVGSHWYATTLQTLL-KYPELLSNP---QRVYW----------
ITYGQTLLIH
KCTD19_MUSMU   PQQIIKLYVGSHWYATTLQTLM-KYPELLSNT---QRVYW----------
IAYGQTLLIH
A4IG48_DANRE   TAERVTLIVDNTRFVVDPSIFT-AQPNTMLGRMFGSGR------
EHNFTRPNEKGEYEVA
X1WDG9_DANRE   TSEKVTLIVDNTRFVVDPSIFT-AQPNTMLGRMFGSGR------
EHNFTRPNEKGEYEVA
BTBDA_MUSMU   TSERVTLIVDNTRFVVDPSIFT-AQPNTMLGRMFGSGR------
EHNFTRPNEKGEYEVA
BTBDA_HOMSA   TSERVTLIVDNTRFVVDPSIFT-AQPNTMLGRMFGSGR------
EHNFTRPNEKGEYEVA
KCTD20_HOMSA   APEKVTLLVDGTRFVVNPQIFT-AHPDTMLGRMFGPGR------
EYNFTRPNEKGEYEIA
KCTD20_MUSMU   APEKVTLLVDGTRFVVNPQIFT-AHPDTMLGRMFGPGR------
EYNFTRPNEKGEYVIA
A0A2R8QE47_DANRE SHDRVTLVVDGTHFVVDPAVFT-AYPDTVLGRMFGRAR------
QHSFTRPNTKGEYEIA
X2J8G3_DROME   PPERITMLVDGVRFTVEQSLLT-AHPTTMLGTMFGSGF--------
QFAHTNERGEYDVA
Q21748_CAEEL   EGDKVCLLVDQTRFLVSQRLLT-SKPDTMLGRMFSMRASCGDLG-
ADLVSPNERDEFEVA
WHI2_SACCE   GDSLIHLNIQENHYFITRDQLM-
SLPESLLLCLFPSGVFLDRCGQVITNLTRDDEVYIVN
WHI2_CANAL   YNSIIHLNIRGKEFTITRDDLM-
SLPESILLCLFPNGVFLDVNGNVINNLTEDDIVY-VN
WHI2_SACPO   DPIIIQLCDRDTVFELSRDQLL-
GLPESILMCLFPRGLLLDYEIQECQLTQRPLIFQTAD

KCTD1_HOMSA   R--DGQMFRYILNFLRTS-
KLLI---------------------------------------
KCTD1_MUSMU   R--DGQMFRYILNFLRTS-
KLLI---------------------------------------
Q5SNS9_DANRE   R--DGHMFRYILNFLRTS-
KLLI---------------------------------------
KCTD15_HOMSA   R--DGEIFRYVLSFLRTS-
KLLL---------------------------------------
KCTD15_MUSMU   R--DGEIFRYILSFLRTS-
KLLL---------------------------------------
Q6DC02_DANRE   R--DGEIFRYILSFLRTC-
KLLL---------------------------------------
Q9W2F9_DROME   R--DGGMFRHILNFMRNS-
RLLI---------------------------------------
KCTD6_HOMSA   R--DGPLFRYVLNFLRTS-

```
ELTL-------------------------------------
KCTD6_MUSMU       R--DGPLFRYVLNFLRTS-
ELTL-------------------------------------
Q6DG99_DANRE      R--DGTLFRYILNFLRTS-
ELTL-------------------------------------
KCTD21_HOMSA      R--DGKVFRYILNFLRTS-
HLDL-------------------------------------
KCTD21_MUSMU      R--DGKVFRYILNFLRTS-
HLDL-------------------------------------
KCTD11_HOMSA      R--DGKAFRHILNFLRLG-
RLDL-------------------------------------
KCTD11_MUSMU      R--DGKAFRHILNFLRLG-
RLDL-------------------------------------
KCTD4_HOMSA       R--DGLLFRHVLNFLRNG-
ELLL-------------------------------------
KCTD4_MUSMU       R--DGLLFRHVLNFLRNG-
ELLL-------------------------------------
Q6GMI6_DANRE      R--DGPIFRHILNFLRLG-
ELVL-------------------------------------
KCTD8_HOMSA       R--DGFLFRYVLDYLRDK-
QLAL-------------------------------------
KCTD8_MUSMU       R--DGFLFRYVLDYLRDK-
QLAL-------------------------------------
Q3L1G3_DANRE      R--DGFLFRYVLDFLRDR-
QLVL-------------------------------------
KCTD16_HOMSA      R--DGFLFRYILDYLRDR-
QVVL-------------------------------------
KCTD16_MUSMU      R--DGFLFRYILDYLRDR-
QVVL-------------------------------------
Q3L1G4_DANRE      R--DGFLFRYVLDYLRDK-
QVVL-------------------------------------
Q3L1G6_DANRE      R--DGFLFRYVLDYLRDK-
TVVL-------------------------------------
Q804Q4_DANRE      R--DGFLFRYILDYLRDL-
NLVL-------------------------------------
Q561T3_DANRE      R--DGFLFRYILDYLRDQ-
TLVL-------------------------------------
KCNRG_MUSMU       R--DGALFSFILDFLRNH-
ELLL-------------------------------------
KCNRG_HOMSA       R--DGDLFSFILDFLRTH-
QLLL-------------------------------------
KCTD12_HOMSA      R--DGFLFRYILDYLRDL-
QLVL-------------------------------------
KCTD12_MUSMU      R--DGFFFRYILDYLRDL-
QLVL-------------------------------------
Q9VDH3_DROME      R--DGVLFRYILDFLRDK-
ALHL-------------------------------------
A0PD34_CAEEL      R--DGPLFAYVLHFLRTD-
KLSL-------------------------------------
KCTD7_HOMSA       R--DGTHFGDVLNFLRSG-
```

```
DLPP-------------------------------------
KCTD7_MUSMU        R--DGTHFGDVLNFLRSG-
DLPP-------------------------------------
Q0VFV7_DANRE       R--DGTYFGDILNFLREG-
ELPQ-------------------------------------
KCTD14_HOMSA       R--PSTYFRPILDYLRTG-
QVPT-------------------------------------
KCTD14_MUSMU       R--PGTYFGLLLDYLRTG-
QVPT-------------------------------------
A0A2R8RRJ7_DANRE   R--DGTLFTHILEYLRTE-
KLPC-------------------------------------
KCTD2_HOMSA        R--DPTYFGPILNYLRHG-
KLII-------------------------------------
Q0P490_DANRE       R--DPTYFGPILNYLRHG-
KLII-------------------------------------
KCTD5_HOMSA        R--DPTYFGPVLNYLRHG-
KLVI-------------------------------------
KCTD5_MUSMU        R--DPTYFGPVLNYLRHG-
KLVI-------------------------------------
Q6NYY3_DANRE       R--DPTYFGPVLNYLRHG-
KLVL-------------------------------------
KCTD2_MUSMU        R--DPTYFGPILNYLRHG-
KLII-------------------------------------
KCTD17_HOMSA       R--DPTYFGPILNFLRHG-
KLVL-------------------------------------
KCTD17_MUSMU       R--DPTYFGPILNFLRHG-
KLVL-------------------------------------
U3JA92_DANRE       R--DPTYFGPILNYLRHG-
KLVY-------------------------------------
Q18776_CAEEL       R--DPDFFSPILNYLRHG-
KLIM-------------------------------------
Q9W579_DROME       R--DPKYFAPVLNYLRHG-
KLVL-------------------------------------
KCTD9_HOMSA        R--SPEYFEPILNYLRHG-
QLIV-------------------------------------
KCTD9_MUSMU        R--SPEYFEPILNYLRHG-
QLIV-------------------------------------
F1Q5M1_DANRE       R--SPEYFEPILNYLRHG-
QIII-------------------------------------
Q6DGD4_DANRE       R--SPDYFEPILNYLRHG-
QLII-------------------------------------
Q8T0F7_DROME       R--SPRYFEPIINYLRHG-
QFVC-------------------------------------
KCTD3_HOMSA        R--DPAAFAPILNFLRTK-
ELDL-------------------------------------
KCTD3_MUSMU        R--DPAAFAPILNFLRTK-
ELDL-------------------------------------
F1Q6W0_DANRE       R--DPTAFAPILNFLRTK-
ELDL-------------------------------------
SHKB1_HOMSA        R--DPTVFAPILNFLRTK-
```

```
ELDP-------------------------------------
SHKB1_MUSMU       R--DPTVFAPILNFLRTK-
ELDP-------------------------------------
E7FFI2_DANRE      R--DPSLFAPILNFLRTK-
ELHP-------------------------------------
Q9VH62_DROME      R--DPTLFSIILNYLRTK-
DIDI-------------------------------------
O17001_CAEEL      R--DPDLFRVILNYLRTK-
QVDL-------------------------------------
Q9TZA6_CAEEL      R--SPKYFETVLNYMRSG-
VTVL-------------------------------------
Q18986_CAEEL      R--DSKHFRLILNFLRDG-
QIAL-------------------------------------
Q7JZ62_DROME      R--CGNHFGIILNYLRDG-
TVPL-------------------------------------
KCTD10_HOMSA      R--CGKHFGTILNYLRDG-
AVPL-------------------------------------
KCTD10_MUSMU      R--CGKHFGTILNYLRDG-
GVPL-------------------------------------
Q6P7X5_DANRE      R--CGKHFGTILNYLRDG-
VVPL-------------------------------------
TNFAIP1_HOMSA     R--CGKHFGTILNYLRDD-
TITL-------------------------------------
TNFAIP1_MUSMU     R--CGKHFGTILNYLRDD-
TITL-------------------------------------
F1R5K5_DANRE      R--CGKHFGSILSYLRDG-
FVNL-------------------------------------
KCTD13_HOMSA      R--SGRHFGTILNYLRDG-
SVPL-------------------------------------
KCTD13_MUSMU      R--SGRHFGTILNYLRDG-
SVPL-------------------------------------
A9ULR9_DANRE      R--CGRHFSLVLNFLRDG-
TVPL-------------------------------------
KCTD18_HOMSA      R--
DGRLFKYLLDYLHGEVQIPT-------------------------------------
KCTD18_MUSMU      R--
DGHLFKYILDYLHGEVQTPS-------------------------------------
KCTD19_HOMSA      G--DGQMFRHILNFLRLG-
KLFL-------------------------------------
KCTD19_MUSMU      G--DGQMFRHILNFLRLG-
KLFL-------------------------------------
A4IG48_DANRE      EGISSTVFRAILDYYKSG-
IIRC-------------------------------------
X1WDG9_DANRE      EGISSTVFRAILDYYKSG-
IIRC-------------------------------------
BTBDA_MUSMU       EGIGSTVFRAILDYYKTG-
IIRC-------------------------------------
BTBDA_HOMSA       EGIGSTVFRAILDYYKTG-
IIRC-------------------------------------
KCTD20_HOMSA      EGISATVFRTVLDYYKTG-
```

```
IINC-------------------------------------
KCTD20_MUSMU      EGISATVFRTVLDYYKTG-
IINC-------------------------------------
A0A2R8QE47_DANRE  EGIGASIFRIILDFYRVG-
ILHC-------------------------------------
X2J8G3_DROME      DGISHLVFRAILEYYKSG-
VIRC-------------------------------------
Q21748_CAEEL      DGMTSSCFRAILDYYQSG-
TMRC-------------------------------------
WHI2_SACCE        F--PPDCFEYIMEIYTKA-
HDDLYNHPVEKFFDRPSSSFVSNAKGFFGLSSNNSISSNNE
WHI2_CANAL        F--DPQTFQYIINTFYQA-QQDLIQMSTNTTNLTPV---
TSHNNGRHNNNNNNSSRHNRQ
WHI2_SACPO        F--DPSLLQYILNYFQMA-
ENRTANDEI---------------------------ALSPP


KCTD1_HOMSA       ---PDDFKDYTLLYEEAKYFQLQPMLLEMER-WKQDRETGRF
KCTD1_MUSMU       ---PDDFKDYTLLYEEAKYFQLQPMLLEMER-WKQDRETGRF
Q5SNS9_DANRE      ---PDDFKDYSLLYEEARYFQLQPLQVELER-WRSEQDSRFT
KCTD15_HOMSA      ---PDDFKDFSLLYEEARYYQLQPMVRELER-WQQEQEQRRR
KCTD15_MUSMU      ---PDDFKDFNLLYEEARYYQLQPMVRELER-WQQDQEQRRR
Q6DC02_DANRE      ---PDDFKDFNLLYEEARYYQLSPMIKELER-WKQEREQRRL
Q9W2F9_DROME      ---AEDFPDLELLLEEARYYEVEPMIKQLES-MRKDRVRNGN
KCTD6_HOMSA       ---PLDFKEFDLLRKEADFYQIEPLIQCLND-PKPLYPMDTF
KCTD6_MUSMU       ---PLDFKEFDLLRKEADFYQIEPLIQCLND-PRPLYPMDTF
Q6DG99_DANRE      ---PVDFTELDLLRKEADFYQIEPLIQCLND-PKPLYPLDTF
KCTD21_HOMSA      ---PEDFQEMGLLRREADFYQVQPLIEALQE-KEVELSKAEK
KCTD21_MUSMU      ---PEDFQEMGLLRREADFYQVQPLIEALQE-KEVELSKAEK
KCTD11_HOMSA      ---PRGYGETALLRAEADFYQIRPLLDALRE-LEASQGTPAP
KCTD11_MUSMU      ---PRGYGETALLKAEADFYQIRPLLDALRE-LEASRGTPAS
KCTD4_HOMSA       ---PEGFRENQLLAQEAEFFQLKGLAEEVKSRWEKEQLTPRE
KCTD4_MUSMU       ---PEGFRENQLLAQEAEFFQLKGLAEEVKSRWEKEQLTPRE
Q6GMI6_DANRE      ---PEDFKETELLRREANFYRLSELAQALQD-WEQQQATQRE
KCTD8_HOMSA       ---PEHFPEKERLLREAEYFQLTDLVKLLSPKVTKQNSLNDE
KCTD8_MUSMU       ---PEHFPEKERLLREAEFFQLTDLVKLLSPKVTKQNSLNDE
Q3L1G3_DANRE      ---PEHFPERERLQREAEHFQLGELLRLLGPRVAKQGSLNDE
KCTD16_HOMSA      ---PDHFPEKGRLKREAEYFQLPDLVKLLTPDEIKQS--PDE
KCTD16_MUSMU      ---PDHFPERGRLKREAEYFQLPDLVKLLAPEDVKQS--PDE
Q3L1G4_DANRE      ---PDHFPEKGRLRKEAEYFQLPDLVKLLTPDDLKPS--SDE
Q3L1G6_DANRE      ---PDYFPEKGRLKREAEFFQLPELVKILTPDDYSHS-DFDE
Q804Q4_DANRE      ---PDYFPEKSRLQREAEFFQLRDLSKLLSPKMSKDNSITDE
Q561T3_DANRE      ---PDYFKEKASLLKEAEYFQLQDLAKRLKPAVSKENSISEE
KCNRG_MUSMU       ---PSDFADHHRLQREALFYELDSLVDLLSQFLLQSRSAVME
KCNRG_HOMSA       ---PTEFSDYLRLQREALFYELRSLVDLLNPYLLQPRPALVE
KCTD12_HOMSA      ---PDYFPERSRLQREAEYFELPELVRRLGAPQQPGPGPPPS
KCTD12_MUSMU      ---PDYFPERSRLQREAEYFELPELVRRLGAPQQPGPGPPPP
Q9VDH3_DROME      ---PEGFRERQRLLREAEHFKLTAMLECIRS---ERDARPPG
A0PD34_CAEEL      ---PEQFREVARLKDEADFYRLERFSTLLSN-ASSISPRPRT
KCTD7_HOMSA       ---RERVRA---VYKEAQYYAIGPLLEQLENMQPLKGEKVRQ
```

```
KCTD7_MUSMU         ---REHVRA---VHKEAQYYAIGPLLEQLENMQPLKGEKVRQ
Q0VFV7_DANRE        ---RDRVRA---VHREAQYYAIGPLLENLEDTQPLTGEKVRQ
KCTD14_HOMSA        -----QHIPE--VYREAQFYEIKPLVKLLEDMPQIFGEQVSR
KCTD14_MUSMU        -----EYVPE--VYQEAKFYQIHLLVKILEDMPQIFGEQVAR
A0A2R8RRJ7_DANRE    -----EHLQE--VHKEAIYYDIKPLVKAIEETPQFFGETVGR
KCTD2_HOMSA         ----TKELAEEGVLEEAEFYNIASLVRLVKERIRDNENRTSQ
Q0P490_DANRE        ----NKNLAEEGVLEEAEFYNIASLVRLVKERIRDNENRTSQ
KCTD5_HOMSA         ----NKDLAEEGVLEEAEFYNITSLIKLVKDKIRERDSKTSQ
KCTD5_MUSMU         ----NKDLAEEGVLEEAEFYNITSLIKLVKDKIRERDSRISQ
Q6NYY3_DANRE        ----NRGLAEEGVLEEAEFYNITSLIKLVKDKIRERDCKTAQ
KCTD2_MUSMU         ----TKELGEEGVLEEAEFYNIASLVRLVKERIRDNENRTSQ
KCTD17_HOMSA        ----DKDMAEEGVLEEAEFYNIGPLIRIIKDRMEEKDYTVTQ
KCTD17_MUSMU        ----DKDMAEEGVLEEAEFYNIGPLIRIIKDRMEEKDYTVAQ
U3JA92_DANRE        ----NKELAEEGVLEEAEFYNITPLIKLIKERILERDSKATQ
Q18776_CAEEL        ----NPGLSEEGILAEADFYNLPSLSQLIMDRIQDRENSVKD
Q9W579_DROME        -----DGVSEEGVLEEAEFYNVTQLIALLKECILHRDQRPQT
KCTD9_HOMSA         ----NDGINLLGVLEEARFFGIDSLIEHLEVAIKNSQPPEDH
KCTD9_MUSMU         ----NDGINLLGVLEEARFFGIDSLIEHLEVAIKNSQPPEDH
F1Q5M1_DANRE        ----NDGINLLGVLEEARFFGIEQLAEQLEVAIKNSHPPEDH
Q6DGD4_DANRE        ----NDGINLLGVLEEARFFGIERLAEQLEGVIKNSQPPDDH
Q8T0F7_DROME        ----DSNISVLGVLEEARFFGIFSLVTHLEERLGQQETPLGD
KCTD3_HOMSA         -----RGVSINVLRHEAEFYGITPLVRRLLLCEELERSSCGS
KCTD3_MUSMU         -----RGVSINVLRHEAEFYGITPLVRRLLLCEELERSSCGS
F1Q6W0_DANRE        -----RGVNISILRHEAEFYGITPLVRRLLLCEELERSSCGS
SHKB1_HOMSA         -----RGVHGSSLLHEAQFYGLTPLVRRLQLREELDRSSCGN
SHKB1_MUSMU         -----RGVHGSSLLHEAQFYGLTPLVRRLQVREELDRSSCGN
E7FFI2_DANRE        -----RSIDVHLLIHEAEFYGITPLVRKLQLCDELDRSSCGN
Q9VH62_DROME        -----KNCEIRALRHEAEYYGITPLTKRLALCEDLNHSSCGD
O17001_CAEEL        -----CGIKVDTLKHEALFFGLTPLIRRLTLCEELSSTSCGS
Q9TZA6_CAEEL        ---PDSEKELQELKKEAEFYLLEQLVDLCEPINNQIRT-YRS
Q18986_CAEEL        ---PDSDREVREVLAEASYFLLDPLIELCGERLEQSLNPYYH
Q7JZ62_DROME        ---PETNKEIAELLAEAKYYCITELAISCERALYAHQ-EPKP
KCTD10_HOMSA        ---PESRREIEELLAEAKYYLVQGLVEECQAALQN-KDTYEP
KCTD10_MUSMU        ---PESRREIEELLAEAKYYLVQGLLEECQAALQN-KDTYEP
Q6P7X5_DANRE        ---PESRRETEELLAEAKYYLVQGLVDECQAALQN-KDAYEP
TNFAIP1_HOMSA       ---PQNRQEIKELMAEAKYYLIQGLVNMCQSALQDKKDSYQP
TNFAIP1_MUSMU       ---PQSRQEIQELMAEAKYYLIQGLVSTCQTALQDKKDSYQP
F1R5K5_DANRE        ---PKSRQSIMELLAEAKYYQIQGLIDLCQKELQDNKEKALC
KCTD13_HOMSA        ---PESTRELGELLGEARYYLVQGLIEDCQLALQQKRETLSP
KCTD13_MUSMU        ---PESARELGELLGEARYYLVQGLIEDCQLALQQKREKLSP
A9ULR9_DANRE        ---PDSTRELEEVLKEAQYYRLQGLVQHCLSTLQKRRDVCRG
KCTD18_HOMSA        ---DEQTRI--ALQEEADYFGIPYPYSLSDHLANE-METYSL
KCTD18_MUSMU        ---DEQTRA--ALQEEADYFGIPYPYSLSDHLANE-METYSL
KCTD19_HOMSA        ---PSEFKEWPLFCQEVEEYHIPSLSEALAQCEAYKSWTQEK
KCTD19_MUSMU        ---PSEFKEWPLFCQEVEEYHIPALSEALAQCEAYKSWTQEK
A4IG48_DANRE        ---PDGISIP-ELREACDYLCIAFDYSTIK-CRDLSALMHEL
X1WDG9_DANRE        ---PDGISIP-ELREACDYLCISFDYSTIK-CRDLSALMHEL
BTBDA_MUSMU         ---PDGISIP-ELREACDYLCISFEYSTIK-CRDLSALMHEL
BTBDA_HOMSA         ---PDGISIP-ELREACDYLCISFEYSTIK-CRDLSALMHEL
KCTD20_HOMSA        ---PDGISIP-DLRDTCDYLCINFDFNTIR-CQDLSALLHEL
```

```
KCTD20_MUSMU       ---PDGISIP-DLRDTCDYLCINFDFNTIR-CQDLSALLHEL
A0A2R8QE47_DANRE   ---PEGVSLA-ELREACDYLCINFDYNTVR-CRDLSALLHEL
X2J8G3_DROME       ---PPTVSVP-ELKEACDYLLIPFDATTVR-CQNLSL-LHEL
Q21748_CAEEL       ---PSSVSVS-ELREACDYLLVPFNAQTVK-CQNLHALLHEL
WHI2_SACCE         QDILHQKPAIIVLREDLDYYCVPQEEFQFDSTNEENNEDLLR
WHI2_CANAL         ENILETKPAIIVLREDLDFYVIPPFERLNS---EQMKQLKLG
WHI2_SACPO         PPSFPGKCGIILLKEDIEFFILPPISPTTNIAIEVSPNDLLK
```
